# External validation of a machine learning model for classifying hospitalization outcomes in Asian elephants across three elephant hospitals in Thailand

**DOI:** 10.1371/journal.pone.0362469

**Published:** 2026-11-11

**Authors:** Worapong Kosaruk, Pichamon Ueangpaibool, Nawapa Hirannithithamrong, Janjeera Kaew-ek, Taweepoke Angkawanish

**Affiliations:** 1 Faculty of Veterinary Medicine, Chiang Mai University, Chiang Mai, Thailand; 2 Elephant, Wildlife and Companion Animals Research Group, Chiang Mai University, Chiang Mai, Thailand; 3 National Elephant Institute, Elephant Hospital, Thai Elephant Conservation Center, Forest Industry Organization, Ministry of Natural Resources and Environment, Lampang, Thailand; 4 National Institute of Elephant Research and Health Service, Division of Animal Welfare and Veterinary Service, Department of Livestock Development, Surin, Thailand; Universidade Federal de Mato Grosso do Sul, BRAZIL

## Abstract

Classification of hospitalization outcomes in wildlife hospitals may support clinical assessment and resource planning, but model performance can vary across institutions with different case profiles and care practices. This study evaluated a previously developed random forest model for classifying hospitalization outcomes in Asian elephants using temporally distinct 2025 data from three elephant hospitals in Thailand. The validation comprised temporal validation at the original development center and institutional validation at two additional hospitals. Because length of stay was included as a predictor, the model was evaluated as a hospitalization-course classification model rather than as an admission-time prognostic tool. The final validation cohort comprised 237 hospitalization episodes contributed by 215 elephants, including 17 deceased, 36 ongoing, and 184 recovered episodes. Overall accuracy was 0.793, compared with 0.776 for a recovered-only majority-class classifier. Macro-averaged balanced accuracy was 0.732, the prevalence-weighted one-vs-rest area under the receiver operating characteristic curve (AUC) was 0.854, and multiclass log loss was 0.617. Classification performance was stronger for ongoing and recovered episodes than for deceased episodes. The limited number of deceased episodes resulted in substantial uncertainty around mortality-focused performance and calibration estimates. Calibration patterns differed across outcome classes, with an overall multiclass Brier score of 0.323. In the binary deceased-versus-recovered sensitivity analysis, the AUC was 0.726 (95% confidence interval, 0.585–0.839), indicating moderate but imprecise discrimination. Overall, the model retained discriminatory information across the validation cohort, but its modest improvement over the majority-class benchmark, weaker deceased-class performance, and reliance on hospitalization-duration information warrant cautious interpretation. Further prospective evaluation is needed before routine clinical application.

## Introduction

Predicting clinical outcomes in wildlife and zoo hospitals presents challenges that differ substantially from those in companion animal practice. Case populations are often highly heterogeneous, encompassing a wide range of species, disease processes, injury mechanisms, and stages of clinical presentation, frequently with limited opportunities for standardized diagnostic workup or longitudinal follow-up [1–3]. In Asian elephants, these challenges are further compounded by long lifespans, the coexistence of chronic and acute health conditions, and the complex physiological and management factors that may influence disease progression and recovery [4,5]. As a result, clinical outcome assessment in elephant hospital settings has implications not only for clinical care, but also for animal welfare, allocation of limited medical resources, and decisions regarding prolonged treatment or end-of-life management. In range countries such as Thailand, where elephant hospitals may operate under resource-constrained conditions while managing high and fluctuating caseloads [6,7], there is a need for more structured and transparent approaches to outcome assessment. Although data-driven prognostic tools have been described in some wildlife rehabilitation settings, such as classification-based models in marine mammals [8], empirically evaluated tools for outcome classification in elephant hospitals remain limited.

Machine learning approaches have increasingly been applied to prognostic modeling across veterinary disciplines, including companion animal practice, livestock systems, equine medicine, and wildlife health [5,9–11]. These methods can integrate multiple clinical, demographic, and management-related variables to estimate the likelihood of outcomes such as mortality, survival, recovery, or workload using routinely collected data [12,13]. In companion animals, machine learning models have been used to predict mortality risk, disease occurrence, and clinical outcomes from structured clinical and laboratory records, with moderate to strong discriminative performance reported in internal validation settings [14–16]. Similar approaches have also been applied in equine and farm animal medicine to support surgical decision-making, estimate survivability, or predict disease events in production systems [17–20]. Within wildlife medicine, applications remain comparatively limited, but include interpretable classification models developed for prognosis and resource allocation in marine mammal rehabilitation [8]. In Asian elephants, recent studies have demonstrated the feasibility of machine learning for both clinical outcome classification and hospital workload forecasting [5,7]. However, most published applications have focused on model development and internal validation within a single institution or time period.

Despite the growth of prognostic modeling in veterinary and wildlife medicine, external validation across different institutions and time periods remains uncommon. Models that perform well during development may show reduced discrimination or poor calibration when applied to new populations with different case mix, disease prevalence, workflows, and resource availability. Evidence from human medicine has shown that prediction models may not maintain performance outside the original development setting, highlighting the importance of external validation across settings and over time [21–24]. In veterinary medicine, and particularly in wildlife or zoo species, formal external validation remains rare. When reported, such evaluations are often limited to single-site or closely related populations [5,8]. This limitation is especially relevant for Asian elephants, in which clinical management occurs across diverse hospital environments and evolving caseloads. Under these conditions, the applicability of a model developed in one institutional setting to broader clinical use cannot be assumed.

In Thailand, elephant hospitals operate within a heterogeneous healthcare landscape that includes referral centers, regional facilities, and field-based hospitals with differing diagnostic capacity, case severity, and management practices [7]. Recently, a machine learning model for clinical outcome classification was developed using retrospective data from a single referral elephant hospital in Thailand, the National Elephant Institute (NEI), based on cases admitted between 2019 and 2024 [5]. That study showed that routinely collected clinical variables could be used to classify clinical outcomes with reasonable discriminative performance in the development setting. However, because the model was derived from a single institution and evaluated primarily through internal validation, its performance in other elephant hospitals and in temporally distinct cohorts remained uncertain.

The objective of this study was to evaluate that previously developed model under both temporal and institutional variation. The validation comprised temporal validation at the original development center using 2025 data and institutional validation at two additional elephant hospitals. Because the original model included length of stay, the present validation was interpreted as an evaluation of hospitalization outcome classification using demographic, diagnostic, and hospitalization-duration information accumulated during care, rather than as validation of prognosis at admission. Specifically, the frozen model was applied without updating or recalibration to hospitalization episodes active during 2025 across three elephant hospitals in Thailand, including the original referral hospital and two additional institutions representing different clinical contexts. Model performance was evaluated using complementary measures of discrimination and calibration within the original three-class outcome framework, together with sensitivity analyses addressing repeated admissions, exclusion of length of stay, and binary deceased-versus-recovered classification.

## Materials and methods

### Study design and data sources

This study externally validated a previously developed machine learning model for classifying clinical outcomes in hospitalized Asian elephants. The original model was developed by Kosaruk et al. [5] using retrospective clinical records collected from a single referral elephant hospital in Thailand, the National Elephant Institute (NEI), between 2019 and 2024. That dataset was used exclusively for model development and internal validation and is described in detail elsewhere.

The external validation dataset consisted of clinical records from hospitalization episodes active during the 2025 calendar year (1 January to 31 December 2025) across three elephant hospitals in Thailand, referred to throughout the manuscript as Hospitals A, B, and C for consistency of presentation. Although one hospital in the validation cohort corresponded to the original development center (Hospital C), no records from the development period were reused. All hospitalization episodes included in the external validation dataset were temporally distinct from the development cohort and were not used at any stage of model development or internal validation. Accordingly, the validation design comprised temporal validation at the original development center (Hospital C) together with institutional validation at Hospitals A and B, which were not represented in the development cohort.

All participating hospitals were government operated and provided both mobile field services and admission-based treatment. Hospital A was located in Surin Province in northeastern Thailand and was operated by the Department of Livestock Development. Its catchment area includes communities with long-standing human-elephant working relationships and a high proportion of privately owned elephants. Hospitals B and C operated under the NEI network and were located in different regions of Thailand. Hospital B was located in Krabi Province in southern Thailand, whereas Hospital C was located in Lampang Province in northern Thailand. Both facilities functioned as regional referral centers for their surrounding areas.

Operational characteristics were described based on information provided by veterinarians at the participating hospitals and differed across hospitals in referral structure, inpatient capacity, and follow-up practices. Hospital A relied more heavily on mobile field-based care and had more limited inpatient capacity, whereas Hospitals B and C functioned as regional referral centers within the NEI network and had greater inpatient capacity. These differences were relevant to the interpretation of ongoing status because active veterinary management at the administrative cutoff did not necessarily imply continued physical hospitalization and could include home-based or mobile follow-up according to local hospital practice.

The aim of the external validation was to assess model performance under temporal and institutional variation by applying the frozen outcome classification model to eligible hospitalization episodes without model refitting, recalibration, or updating.

### Data inclusion and predictor variables

Clinical records were eligible for inclusion if they represented a hospitalization episode active at any time during the 2025 calendar year and contained a clearly documented admission date, elephant age, sex, primary disease category, and clinical outcome status. A discharge date was required for episodes that had concluded, whereas a missing discharge date was not an exclusion criterion for episodes classified as ongoing because length of stay for these episodes was administratively censored at 31 December 2025.

Clinical outcome status was defined at the administrative cutoff of 31 December 2025. Episodes were classified as: 1) deceased, if death occurred during the hospitalization episode; 2) recovered, if the episode had concluded following clinical recovery; and 3) ongoing, if the episode had not yet been classified as recovered or deceased and the elephant remained under active veterinary management at the administrative cutoff. Ongoing status did not necessarily indicate continued physical hospitalization and could include continued home-based or mobile follow-up according to local hospital practice. The validation dataset was extracted on 29 January 2026. During manual record verification, outcome status was reviewed using the same operational definition across all three hospitals with reference to the 31 December 2025 administrative cutoff. For ongoing episodes, length of stay was administratively censored at 31 December 2025. Subsequent clinical resolution after the administrative cutoff was not used to relabel outcome status in the validation dataset. Clinical outcomes occurring after the data extraction date were not systematically ascertained.

Length of stay was generated during hospitalization and was therefore not available at admission. This variable may capture information arising during the clinical course and may also reflect discharge practices, inpatient capacity, follow-up arrangements, ongoing-care definitions, and the administrative timing of outcome ascertainment. For ongoing episodes, length of stay was administratively censored at 31 December 2025 and was therefore additionally linked to the prespecified administrative cutoff used to define outcome status. Accordingly, the primary model incorporates information accumulated during hospitalization and should be interpreted as a hospitalization-course classification model rather than an admission-time prognostic tool. Length of stay was retained in the primary analysis to preserve external validation of the frozen model and its original predictor set.

Predictor variables were specified to match those used in the original model developed by Kosaruk et al. [5]. These predictors included patient age in years, sex, primary disease category, and length of stay in days. Age and length of stay were treated as continuous variables, whereas sex and disease category were treated as categorical variables.

Disease categories in the external validation dataset were harmonized using a predefined mapping derived from the development cohort. Categories represented by fewer than five cases in the development dataset were grouped into an Other category, with the exception of Miscellaneous, which was retained as a separate category. This mapping and its factor levels were defined from the development cohort before external validation and then applied unchanged to the external dataset to avoid data-driven category redefinition.

The unit of analysis was the hospitalization episode. Elephants with more than one eligible hospitalization during the study period could therefore contribute multiple episodes to the primary analysis. Repeated admissions by the same elephant were retained because each represented a separate hospitalization episode.

### Data preprocessing and model application

All preprocessing procedures used during model development were defined by the frozen preprocessing recipe derived from the development dataset and applied unchanged to the external validation data. Factor levels and the corresponding one-hot encoded dummy-variable structure were therefore determined from the development data. Zero-variance predictor removal was also part of the development preprocessing recipe and was not recalculated using the external validation dataset. No additional feature engineering, scaling, imputation, or data-driven preprocessing was introduced during external validation.

The classification model was a random forest classifier implemented using the ranger package in R, as described previously [5]. The final fitted model, including its hyperparameter settings and class-weighting structure, was loaded from the stored model object and applied directly to the externally preprocessed validation dataset. Predicted class probabilities were generated for each outcome category, and the classified outcome was defined as the category with the highest predicted probability.

### Performance evaluation

Model performance was evaluated using measures of discrimination and calibration. Overall multiclass performance was summarized using classification accuracy, macro-averaged balanced accuracy, prevalence-weighted one-vs-rest area under the receiver operating characteristic curve (AUC), and multiclass log loss. The prevalence-weighted one-vs-rest AUC was calculated as the weighted mean of the three class-specific AUCs, using the observed prevalence of each outcome class as the weight. Agreement between observed and classified outcomes was further summarized using a confusion matrix. To contextualize overall classification accuracy in the presence of class imbalance, model accuracy was compared with a majority-class benchmark that classified every hospitalization episode as recovered, the most prevalent outcome in the validation cohort.

Discrimination for each outcome category was assessed using one-versus-rest receiver operating characteristic curves. Calibration was assessed separately for each outcome class using a one-vs-rest framework. Graphical calibration was evaluated by comparing mean predicted probabilities with observed event rates across deciles of predicted probability, with exact 95% binomial confidence intervals for the observed event rates. Quantitative calibration was summarized using the Brier score, scaled Brier score, calibration-in-the-large, and calibration slope. Overall probabilistic performance was additionally summarized using the multiclass Brier score. Ninety-five percent confidence intervals for the quantitative calibration measures and multiclass Brier score were estimated using 2,000 bootstrap resamples clustered by elephant. No post hoc recalibration was performed.

Performance metrics were also summarized separately for each participating hospital to explore possible heterogeneity in model performance across institutions. These analyses were descriptive and were interpreted in relation to outcome distribution and sample size within each hospital.

Per-class performance metrics, including precision, recall, specificity, F1-score, and balanced accuracy, were calculated using a one-versus-rest framework. Ninety-five percent confidence intervals were estimated using 2,000 bootstrap resamples clustered by elephant to account for repeated hospitalization episodes contributed by the same individual. To assess the influence of repeated admissions on the primary findings, a sensitivity analysis was performed after retaining only the first eligible hospitalization episode for each elephant. Model performance in this first-episode-only cohort was compared with that of the complete episode-level cohort.

Descriptive performance summaries were additionally generated by disease category for subgroups with at least 15 hospitalization episodes. Balanced accuracy and the prevalence-weighted one-vs-rest AUC could not be estimated for certain disease categories because one or more outcome classes were absent within those subgroups. These analyses were conducted to characterize patterns of model behavior rather than to support formal statistical inference.

To examine the dependence of classification performance on length of stay, a separate exploratory random forest model was fitted using the original development dataset but restricted to age, sex, and primary disease category. This model was then evaluated in the same external validation cohort. Because the model was fitted separately after removal of a predictor, this analysis was considered exploratory and did not represent external validation of the frozen primary model.

A binary deceased-versus-recovered sensitivity analysis was performed after excluding ongoing episodes. The deceased-outcome probabilities generated by the frozen multiclass model were evaluated without model refitting or recalibration. Discrimination was assessed using the AUC, with 95% confidence intervals estimated using 2,000 bootstrap resamples clustered by elephant. Graphical calibration was evaluated by comparing mean predicted probabilities of the deceased outcome with observed event rates across deciles of predicted probabilities, with exact 95% binomial confidence intervals for the observed event rates. Quantitative calibration was additionally summarized using the Brier score, scaled Brier score, calibration-in-the-large, and calibration slope.

### Statistical software

All analyses were performed using R statistical software (version 4.5.1). Data preprocessing and model application were implemented using the tidymodels framework, and the random forest model was applied using the ranger package. Performance metrics and visualizations were generated using yardstick, pROC, and ggplot2. Bootstrap procedures were implemented using custom resampling functions.

### Ethical statement

Ethical review and approval were waived for this study because it involved retrospective analysis of existing clinical records generated during routine veterinary care. This project complied with institutional and national regulations governing the use of retrospective clinical data. Clinical information was obtained from participating elephant hospitals under the National Elephant Institute and the National Institute of Elephant Research and Health Service. No additional animal intervention, handling, or sample collection was performed for the purposes of this study.

## Results

### External validation cohort

Across the three hospitals, 243 source records were screened, including 33 from Hospital A, 84 from Hospital B, and 126 from Hospital C. Four source records from Hospital A were excluded because they did not represent independent clinical admissions. During subsequent record-level verification, one record from Hospital B was excluded because of an unresolved date inconsistency, and one duplicate hospitalization record from Hospital C was removed. The final external validation cohort therefore comprised 237 hospitalization episodes, including 29 from Hospital A, 83 from Hospital B, and 125 from Hospital C, contributed by 215 elephants. Twenty-two elephants contributed repeated admissions, accounting for 44 episodes (Figure S1 in S1 File). Baseline demographic and clinical characteristics are summarized in Table 1. Case mix differed across hospitals, with Hospital A including a younger population and a shorter median length of stay than Hospitals B and C. Gastrointestinal conditions were the most common disease category overall, followed by integumentary, eye, and musculoskeletal conditions. Outcome distributions differed across hospitals, with ongoing episodes concentrated at Hospital C and relatively few ongoing episodes at Hospitals A and B. Deceased episodes were uncommon overall (Table 1).

**Table 1:** Baseline characteristics of the external validation cohort. Demographic and clinical characteristics of 237 hospitalization episodes contributed by 215 elephants and included in the 2025 external validation cohort, shown overall and stratified by hospital. Continuous variables are presented as median [interquartile range, IQR], and categorical variables as n (%). Disease categories were harmonized using the predefined development-cohort mapping. Length of stay for ongoing episodes was administratively censored at 31 December 2025.

Characteristic		Overall (N = 237)	A (N = 29)	B (N = 83)	C (N = 125)
Age (years), median [IQR]		30.0 [13.0, 48.0]	15.0 [7.0, 24.0]	35.0 [17.5, 47.5]	34.0 [15.0, 50.0]
Sex, n (%)	Female	128 (54.0)	11 (37.9)	45 (54.2)	72 (57.6)
	Male	109 (46.0)	18 (62.1)	38 (45.8)	53 (42.4)
Disease, n (%)	Integument	47 (19.8)	7 (24.1)	9 (10.8)	31 (24.8)
	Gastrointestinal	69 (29.1)	7 (24.1)	34 (41.0)	28 (22.4)
	Eye	35 (14.8)	4 (13.8)	14 (16.9)	17 (13.6)
	Musculoskeletal	33 (13.9)	5 (17.2)	12 (14.5)	16 (12.8)
	Miscellaneous	18 (7.6)	0 (0.0)	2 (2.4)	16 (12.8)
	EEHV	16 (6.8)	4 (13.8)	8 (9.6)	4 (3.2)
	Reproductive	6 (2.5)	0 (0.0)	2 (2.4)	4 (3.2)
	Toxicity	1 (0.4)	0 (0.0)	0 (0.0)	1 (0.8)
	Dental/Oral	5 (2.1)	1 (3.4)	0 (0.0)	4 (3.2)
	Urinary/Renal	1 (0.4)	0 (0.0)	1 (1.2)	0 (0.0)
	Other	6 (2.5)	1 (3.4)	1 (1.2)	4 (3.2)
Length of stay (days), median [IQR]		14 [6, 52]	7 [4, 14]	10 [6, 34]	21 [8, 116]
Outcome, n (%)	Deceased	17 (7.2)	2 (6.9)	4 (4.8)	11 (8.8)
	Ongoing	36 (15.2)	3 (10.3)	3 (3.6)	30 (24.0)
	Recovered	184 (77.6)	24 (82.8)	76 (91.6)	84 (67.2)

EEHV, elephant endotheliotropic herpesvirus

### Overall multiclass classification performance

Agreement between observed and classified outcomes is shown in the confusion matrix in Fig 1, while class-specific discrimination is presented in Fig 2. Overall accuracy was 0.793, only modestly higher than the recovered-only majority-class benchmark of 0.776, corresponding to an absolute accuracy gain of 0.017. Macro-averaged balanced accuracy was 0.732, and performance differed substantially across outcome classes, with lower precision, recall, and F1-score for deceased episodes than for ongoing and recovered episodes (Table 2). Among the 17 deceased episodes, precision was 0.207 (95% CI, 0.069–0.367), recall was 0.353 (95% CI, 0.133–0.588), and the F1-score was 0.261 (95% CI, 0.097–0.421) (Table 2, panel B). Hospital-specific performance varied across the three validation settings, with the greatest discrimination and lowest probabilistic error observed at Hospital B, whereas Hospital A showed the lowest balanced accuracy and the highest log loss (Table S1 in S1 File).

**Table 2:** Overall and class-specific performance of the frozen random forest classification model in the external validation cohort. Class-specific performance measures were calculated using one-vs-rest definitions; 95% confidence intervals were estimated using 2,000 bootstrap resamples clustered by elephant. The majority-class benchmark classified all hospitalization episodes as recovered.

Panel A. Overall model performance					
Performance measure	Estimate				
Hospitalization episodes, n	237				
Unique elephants, n	215				
Overall accuracy	0.793				
Recovered-only majority-class accuracy	0.776				
Absolute accuracy gain	0.017				
Macro-averaged balanced accuracy	0.732				
Prevalence-weighted one-vs-rest AUC	0.854				
Multiclass log loss	0.617				
Multiclass Brier score	0.323				
**Panel B.** Class-specific performance					
**Outcome class**	**Support, n**	**Balanced accuracy** **(95% CI)**	**Precision** **(95% CI)**	**Recall/Sensitivity** **(95% CI)**	**F1-score** **(95% CI)**
Deceased	17	0.624 (0.513-0.743)	0.207 (0.069-0.367)	0.353 (0.133-0.588)	0.261 (0.097-0.421)
Ongoing	36	0.813 (0.735-0.887)	0.750 (0.591-0.886)	0.667 (0.500-0.815)	0.706 (0.575-0.812)
Recovered	184	0.760 (0.692-0.825)	0.898 (0.852-0.940)	0.859 (0.805-0.908)	0.878 (0.840-0.912)

AUC, area under the receiver operating characteristic curve; CI, confidence interval.

**Fig 1:**
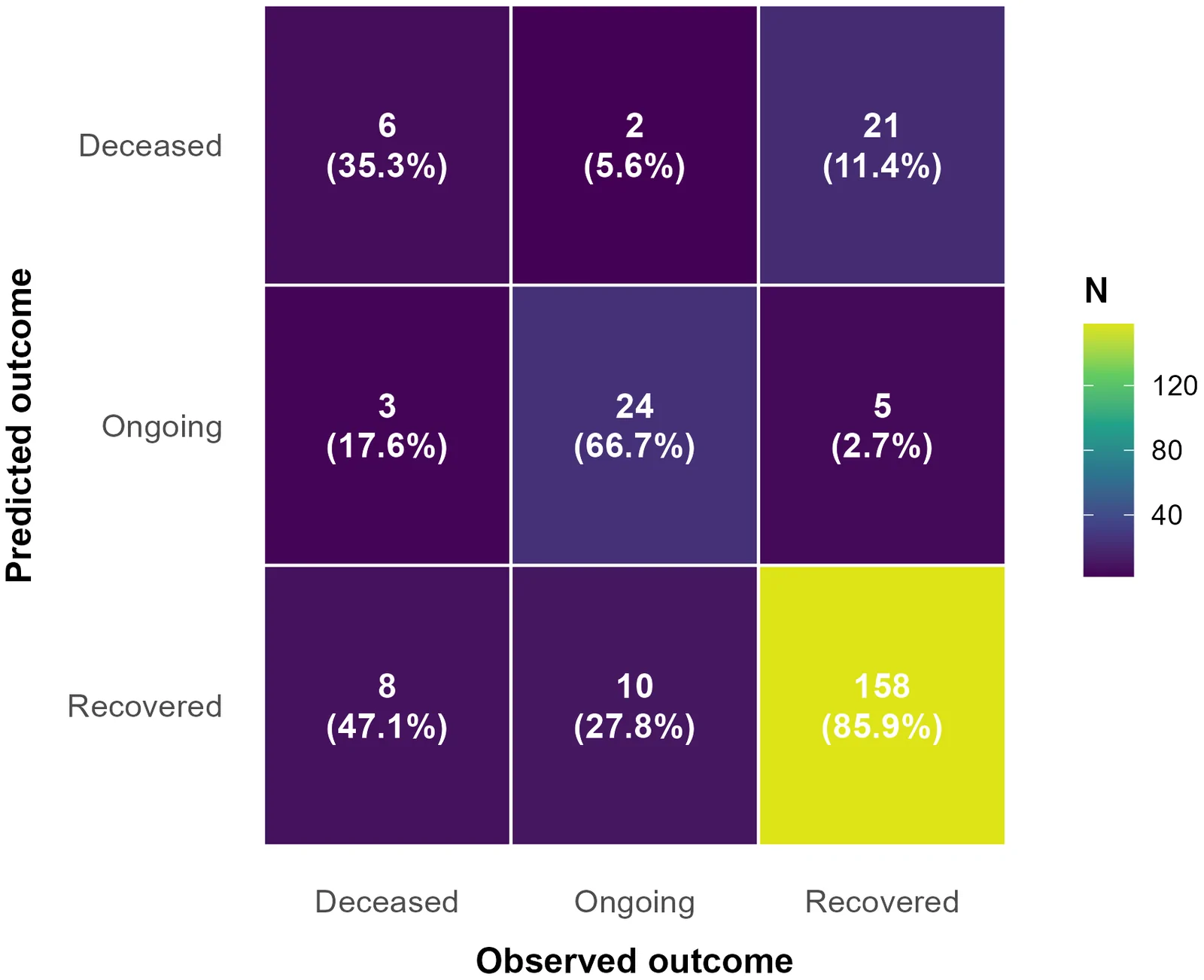
Confusion matrix for multiclass hospitalization outcome classification in the external validation cohort. Agreement between observed hospitalization outcomes and model-classified outcomes across the three hospitals. Values indicate the number of hospitalization episodes, with percentages calculated within each observed outcome category.

**Fig 2:**
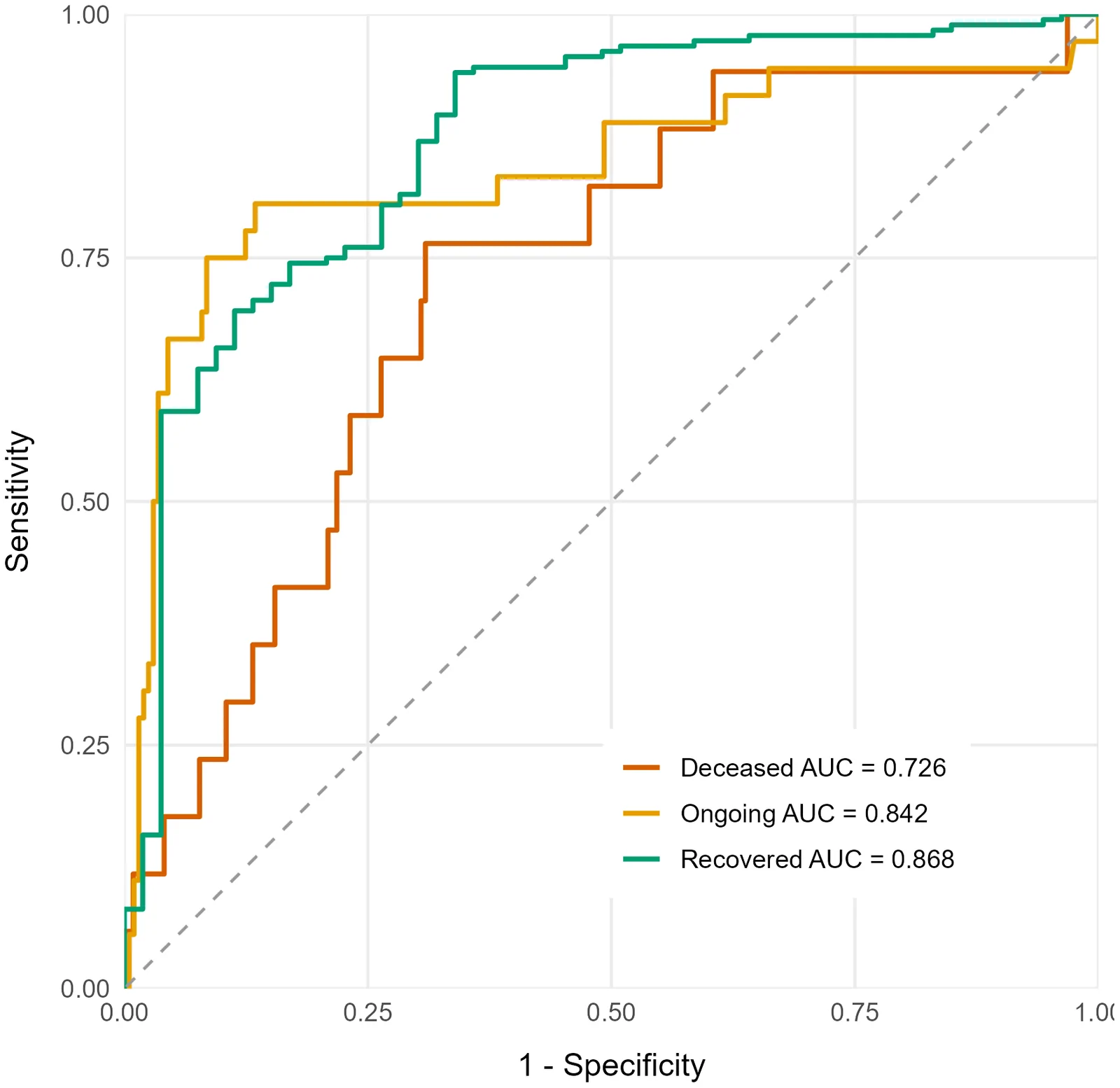
Discrimination performance of the multiclass hospitalization outcome classification model. One-vs-rest receiver operating characteristic (ROC) curves for deceased, ongoing, and recovered hospitalization outcomes in the external validation cohort. AUC, area under the receiver operating characteristic curve.

### Calibration of predicted probabilities

Calibration patterns differed across outcome classes. Calibration-in-the-large was −1.594 (95% CI, −2.325 to −1.081) for deceased, −0.195 (95% CI, −0.673 to 0.212) for ongoing, and 1.131 (95% CI, 0.810 to 1.500) for recovered outcomes (Fig 3; Table S2 in S1 File). Calibration slopes were 0.635 (95% CI, 0.239–1.042), 0.864 (95% CI, 0.571–1.280), and 1.164 (95% CI, 0.873–1.594), respectively. Uncertainty was greatest for the deceased class, consistent with the limited number of deceased episodes. The overall multiclass Brier score was 0.323 (95% CI, 0.271–0.377).

**Fig 3:**
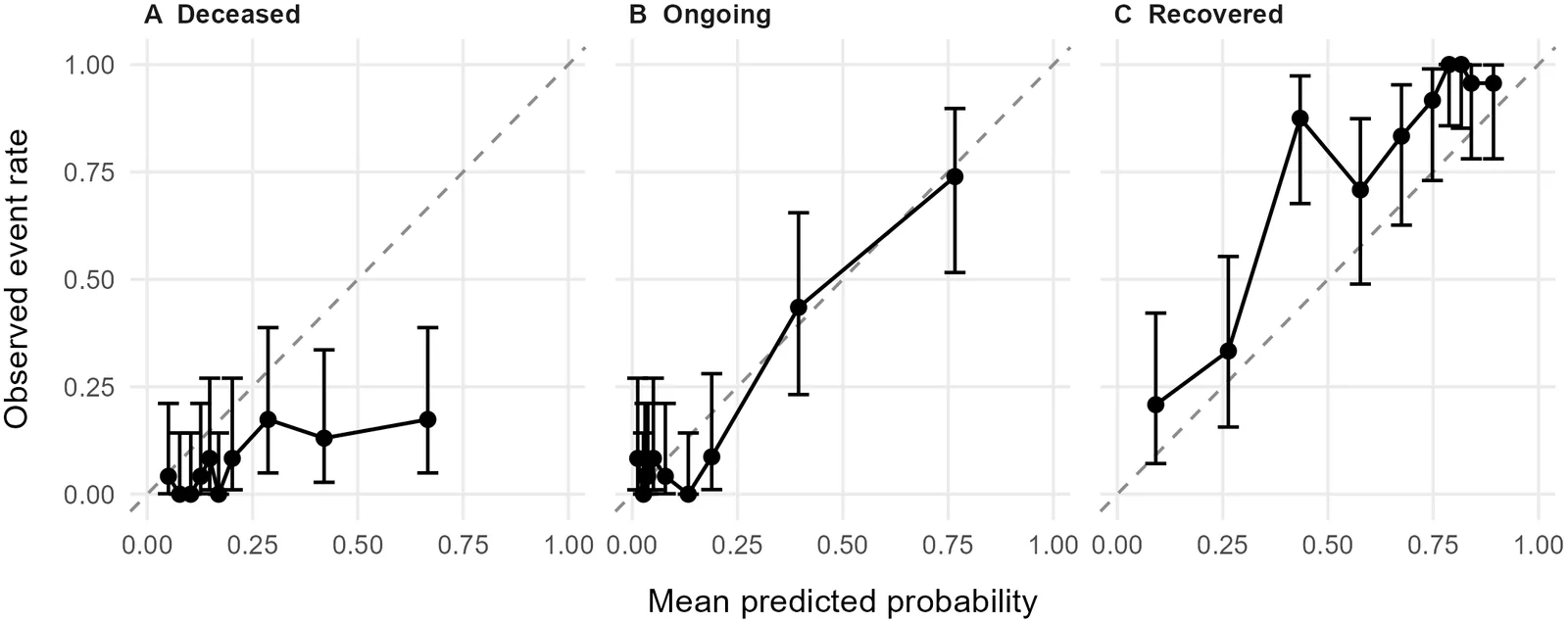
Calibration of predicted probabilities by hospitalization outcome class. Calibration plots comparing mean predicted probabilities with observed event rates for (A) deceased, (B) ongoing, and (C) recovered outcomes in the external validation cohort. Predictions were grouped into deciles of predicted probability. Error bars indicate exact 95% binomial confidence intervals, and the dashed line indicates perfect calibration.

### Disease-stratified performance

Exploratory performance stratified by primary disease category is presented in Table S3 in S1 File for disease groups containing at least 15 hospitalization episodes. Performance estimates varied across disease categories, but several subgroups contained few or no observations for one or more outcome classes. These analyses were considered descriptive and exploratory and were not used to infer disease-specific model validity.

### Sensitivity analyses

Restricting the analysis to the first eligible hospitalization episode per elephant produced performance estimates similar to those of the complete episode-level cohort (Table S4A in S1 File). In contrast, the exploratory model excluding length of stay showed lower overall discrimination and balanced accuracy and did not classify any episodes as ongoing (Table S4B in S1 File). In the binary deceased-versus-recovered analysis, discrimination was moderate, with an AUC of 0.726 (95% CI, 0.585–0.839). The wide confidence interval indicated substantial uncertainty associated with the limited number of deceased episodes (Table S5 in S1 File; Figure S2 in S1 File).

## Discussion

This study evaluated a previously developed random forest model for classifying hospitalization outcomes in Asian elephants using temporally distinct 2025 data from three elephant hospitals in Thailand. The frozen model was applied without refitting, recalibration, or updating, comprising temporal validation at the original development center and institutional validation at two additional hospitals. The model retained discriminatory information at the cohort level, but overall accuracy was only modestly higher than the recovered-only majority-class benchmark, and performance varied across outcome classes and hospitals. Classification performance was stronger for ongoing and recovered episodes than for deceased episodes, for which estimates were imprecise because only 17 deceased episodes were available. Calibration patterns also differed across outcome classes. These findings indicate that model performance was context dependent and should be interpreted cautiously, particularly for mortality-related classification.

The overall preservation of model performance is notable because attenuation in discrimination and shifts in calibration are commonly observed when prediction models are evaluated outside their original development setting. In both veterinary and human medicine, models that perform well during development often show reduced performance when applied to later time periods or different institutions because of variation in case mix, outcome prevalence, workflows, and resource availability [18,22,23,25,26]. The present study therefore adds evidence from an underrepresented clinical domain by examining model behavior across temporally distinct data and multiple elephant hospitals. This is important because predictive tools intended for clinical use should be evaluated under heterogeneous conditions before broader implementation is considered [15,16,27].

Across the multiclass outcome framework, model performance differed by outcome category, with stronger classification performance for recovered and ongoing episodes than for deceased episodes. The deceased outcome was the least frequent class in the external validation cohort, which limited the precision of mortality-focused performance estimates. In addition to class imbalance, the predictor set may have been clinically insufficient for mortality classification. The model included only age, sex, broad primary disease category, and length of stay and therefore did not capture potentially important mortality-related information such as disease severity, physiological status, referral delay, treatment intensity, comorbidities, or clinician-assessed prognosis. These unmeasured dimensions may contribute to heterogeneity among deceased episodes and may partly explain the lower performance observed for this class. Mortality-related classification should therefore be interpreted cautiously, and future models may require richer clinical or longitudinal information to distinguish deceased outcomes more reliably [28].

Institutional context likely also contributed to the observed variation in performance. The participating hospitals differed not only in referral structure and resource availability, but also in admission pathways, inpatient capacity, follow-up practices, and the operational meaning of ongoing care. Hospital A functioned primarily as a primary care service, with cases commonly entering through mobile field assessment and relatively few cases described as emergency or critical. It also had limited inpatient capacity and a predominantly mild-to-moderate admitted case mix, which is consistent with the younger population, shorter length of stay, and relatively small number of ongoing episodes observed in the analytic cohort. By contrast, Hospitals B and C operated within the NEI network and managed a broader mix of primary and referral cases. Hospital C had a substantially higher proportion of ongoing episodes, whereas Hospitals A and B each contributed only three ongoing episodes. Importantly, ongoing status represented active veterinary management at the administrative cutoff and did not necessarily indicate continued physical hospitalization; in some settings, it also included home-based or mobile follow-up. In addition, local workflow constraints, including stall availability, staffing limitations, and the need to shorten hospitalization during periods of high caseload, may have influenced discharge timing and observed outcome distribution. In prognostic research, such differences are consistent with spectrum effects, whereby model performance changes across populations with different baseline risk, case composition, and care pathways [15,29]. The between-hospital differences observed here therefore likely reflect expected contextual variation rather than instability of the model alone.

Calibration analysis provided complementary information to discrimination, but the findings did not support a uniform conclusion of good calibration across outcome classes. Quantitative calibration measures indicated overall overestimation of deceased-outcome probabilities and underestimation of recovered-outcome probabilities, whereas calibration for the ongoing class was closer to the ideal values. The deceased class also showed poorer probabilistic performance than the prevalence-based reference model. Because only 17 deceased episodes were available, both the graphical calibration pattern and quantitative estimates for this class should be interpreted cautiously. This distinction is important because many machine learning studies in veterinary and biomedical medicine emphasize discrimination metrics while giving less attention to calibration [14,30,31]. However, discrimination alone does not indicate whether predicted probabilities are numerically aligned with observed outcomes [16,22]. Accordingly, the predicted probabilities in the present study should not be regarded as clinically calibrated estimates without further validation and, where appropriate, recalibration.

Performance also varied across hospitals and disease categories. These findings should be interpreted primarily as reflections of local data structure rather than as evidence that the model was inherently more valid in some subgroups than others. The hospital-specific analyses were descriptive and were influenced by sample size, outcome prevalence, and subgroup composition. Similarly, disease-stratified analyses were exploratory and were limited by sparse events in several disease categories, which resulted in undefined balanced accuracy and related metrics when one or more outcome classes were absent. Such limitations are common in subgroup analyses of veterinary and biomedical machine learning studies when event counts are low or class imbalance is pronounced [17,18]. The subgroup findings are therefore best interpreted as contextual summaries of model behavior rather than as formal evidence of differential classification performance.

The binary deceased-versus-recovered sensitivity analysis provided a complementary assessment after ongoing episodes were excluded. Discrimination was moderate, but the wide confidence interval reflected substantial uncertainty associated with the limited number of deceased episodes. Calibration measures also indicated overestimation of deceased-outcome probabilities. Accordingly, this analysis should be interpreted cautiously as a sensitivity analysis rather than as evidence of stable mortality classification across alternative outcome formulations.

Several limitations should be considered. First, the number of deceased cases in the external dataset was relatively small, which reduced statistical precision for mortality-focused analyses and widened uncertainty around class-specific performance estimates. Second, recovered episodes constituted the majority of the validation cohort, and overall accuracy was only modestly higher than that of the recovered-only majority-class benchmark. Overall accuracy should therefore be interpreted together with balanced accuracy, class-specific performance, probabilistic error, and calibration rather than as a standalone measure of model performance. Third, twenty-two elephants contributed more than one eligible hospitalization episode, accounting for 44 episodes, so episode-level observations were not completely independent. To account for this clustering, confidence intervals were estimated using bootstrap resampling clustered by elephant, and a sensitivity analysis retaining only the first eligible hospitalization episode per elephant produced similar overall performance estimates. Nevertheless, some within-elephant dependence may remain in the episode-level analysis. Fourth, the ongoing category reflects a dynamic clinical and administrative status rather than a final endpoint, and its interpretation may differ across hospitals depending on workflow, monitoring practices, follow-up arrangements, and discharge timing. Fifth, although the validation dataset incorporated new institutions, one participating hospital was also the original development center, albeit from a later time period. Accordingly, this study represents external validation under temporal shift and partial institutional variation rather than a fully independent multi-institutional validation in which all centers are entirely external to model development. Sixth, the frozen model included length of stay, which is generated during hospitalization and may reflect not only the patient’s clinical course but also discharge practices, inpatient capacity, follow-up arrangements, ongoing-care definitions, and the administrative timing of outcome ascertainment. Retaining this variable was necessary to preserve the original predictor set during external validation, but its inclusion limits interpretation of the model as an admission-time prognostic tool. In the exploratory analysis, the model excluding length of stay showed lower overall discrimination and balanced accuracy and did not classify any ongoing episodes. Although this separately fitted model was exploratory, these findings suggest that hospitalization-duration information contributed to classification performance. Finally, the model was applied without recalibration or updating. This preserves the methodological purpose of external validation, but the potential effects of local recalibration or model updating were not evaluated in the present study.

Taken together, these findings indicate that machine learning-based hospitalization outcome classification warrants further evaluation in elephant hospital settings but does not yet establish clinical utility. The observed class-specific performance and calibration differences, together with the influence of hospitalization-duration and workflow-related information, limit direct clinical interpretation of the current model. Prospective evaluation is needed to assess performance under routine use, examine the effects of local recalibration or model updating, and determine whether model outputs can be incorporated safely and meaningfully into elephant hospital workflows.

## Conclusion

This study evaluated a previously developed frozen random forest model for classifying hospitalization outcomes in Asian elephants through temporal validation at the original development center and institutional validation at two additional elephant hospitals in Thailand. The model retained discriminatory information in the 2025 validation cohort, but overall accuracy was only modestly higher than the recovered-only majority-class benchmark, and classification performance was stronger for ongoing and recovered episodes than for deceased episodes. Because length of stay was included as a predictor, the model should be interpreted as a hospitalization-course classification model using information accumulated during care rather than as an admission-time prognostic tool. The limited number of deceased episodes and the observed class-specific calibration differences further warrant cautious interpretation. These findings do not establish readiness for routine clinical use and support further prospective evaluation, with local recalibration or model updating considered where appropriate before implementation.

## Supporting information

S1 File Supplementary tables and figures. (DOCX)
