# Supplementary material for: External validation of a machine learning model for classifying hospitalization outcomes in Asian elephants across three elephant hospitals in Thailand
Source: PLoS One. 2026 Nov 11;21(11):e0362469. doi: 10.1371/journal.pone.0362469 (PMC13754164; doi:10.1371/journal.pone.0362469)
Supplement: Supplementary file 1 [file pone.0362469.s001.docx]

**Supplementary Table S1. Hospital-specific performance of the frozen random forest classification model in the external validation cohort.** Balanced accuracy was macro-averaged across the three outcome classes.

| **Hospital** | **N** | **Outcome count** | | | **Accuracy** | **Balanced accuracy (macro)** | **Prevalence-weighted one-vs-rest AUC** | **Multiclass log loss** | **Multiclass Brier score** |
| --- | --- | --- | --- | --- | --- | --- | --- | --- | --- |
|  |  | **Deceased,**  **n (%)** | **Ongoing,**  **n (%)** | **Recovered,**  **n (%)** |  |  |  |  |  |
| A | 29 | 2 (6.9) | 3 (10.3) | 24 (82.8) | 0.759 | 0.632 | 0.697 | 0.917 | 0.402 |
| B | 83 | 4 (4.8) | 3 (3.6) | 76 (91.6) | 0.819 | 0.738 | 0.893 | 0.493 | 0.269 |
| C | 125 | 11 (8.8) | 30 (24.0) | 84 (67.2) | 0.784 | 0.737 | 0.862 | 0.629 | 0.341 |

AUC, area under the receiver operating characteristic curve.

**Supplementary Table S2. Quantitative calibration measures of the frozen random forest classification model in the external validation cohort.** Calibration was evaluated separately for each outcome class using a one-vs-rest approach. The 95% CIs were estimated using 2,000 bootstrap resamples clustered by elephant. A scaled Brier score of 0 represents performance equivalent to the prevalence-based reference model; ideal values are 0 for calibration-in-the-large and 1 for calibration slope.

**Panel A.** Class-specific calibration

| **Outcome class** | **Events, n (%)** | **Brier score (95% CI)** | **Scaled Brier score (95% CI)** | **Calibration-in-the-large (95% CI)** | **Calibration slope (95% CI)** |
| --- | --- | --- | --- | --- | --- |
| Deceased | 17 (7.2) | 0.102 (0.081 to 0.126) | −0.538 (−1.467 to −0.138) | −1.594 (−2.325 to −1.081) | 0.635 (0.239 to 1.042) |
| Ongoing | 36 (15.2) | 0.081 (0.057 to 0.107) | 0.371 (0.176 to 0.528) | −0.195 (−0.673 to 0.212) | 0.864 (0.571 to 1.280) |
| Recovered | 184 (77.6) | 0.140 (0.118 to 0.164) | 0.194 (−0.019 to 0.355) | 1.131 (0.810 to 1.500) | 1.164 (0.873 to 1.594) |

**Panel B.** Overall probabilistic performance

| **Measure** | **Estimate (95% CI)** |
| --- | --- |
| Multiclass Brier score | 0.323 (0.271 to 0.377) |

CI, confidence interval.

**Supplementary Table S3. Exploratory performance of the frozen random forest classification model stratified by primary disease category.** Analyses were exploratory and restricted to disease categories with ≥15 hospitalization episodes. Balanced accuracy and prevalence-weighted one-vs-rest AUC were not reported when one or more outcome classes were absent. Confidence intervals were not calculated because of sparse events within several subgroups.

| **Primary disease category** | **Hospitalization episodes, n** | **Outcome count** | | | **Accuracy** | **Balanced accuracy (macro)** | **Prevalence-weighted one-vs-rest AUC** | **Multiclass log loss** |
| --- | --- | --- | --- | --- | --- | --- | --- | --- |
|  |  | **Deceased, n** | **Ongoing, n** | **Recovered, n** |  |  |  |  |
| Eye | 35 | 0 | 2 | 33 | 0.971 | NA | NA | 0.371 |
| Gastrointestinal | 69 | 1 | 0 | 68 | 0.913 | NA | NA | 0.336 |
| Integument | 47 | 2 | 8 | 37 | 0.851 | 0.682 | 0.872 | 0.530 |
| Miscellaneous | 18 | 0 | 11 | 7 | 0.833 | NA | NA | 0.506 |
| Musculoskeletal | 33 | 6 | 9 | 18 | 0.697 | 0.662 | 0.792 | 0.806 |
| EEHV | 16 | 2 | 2 | 12 | 0.188 | 0.526 | 0.797 | 1.611 |

AUC, area under the receiver operating characteristic curve; EEHV, elephant endotheliotropic herpesvirus; NA, not estimable.

**Supplementary Table S4A. Sensitivity analysis restricted to the first eligible hospitalization episode per elephant.** The sensitivity analysis retained only the first eligible hospitalization episode for each elephant. The frozen model was applied without refitting, recalibration, or updating.

| **Analysis** | **Hospitalization episodes, n** | **Elephants, n** | **Accuracy** | **Balanced accuracy (macro)** | **Prevalence-weighted one-vs-rest AUC** | **Multiclass log loss** | **Multiclass Brier score** |
| --- | --- | --- | --- | --- | --- | --- | --- |
| All eligible hospitalization episodes | 237 | 215 | 0.793 | 0.732 | 0.854 | 0.617 | 0.323 |
| First eligible episode per elephant | 215 | 215 | 0.800 | 0.752 | 0.869 | 0.607 | 0.318 |

AUC, area under the receiver operating characteristic curve.

**Supplementary Table S4B. Exploratory sensitivity analysis excluding length of stay from the predictor set.** The exploratory model was fitted separately using the original development dataset with age, sex, and primary disease category as predictors and was evaluated in the same external validation cohort. It did not represent external validation of the frozen primary model after removal of a predictor. No episodes were classified as ongoing; therefore, precision and F1-score for this class were undefined.

**Panel A.** Overall model performance

| **Analysis** | **Hospitalization episodes, n** | **Elephants, n** | **Accuracy** | **Balanced accuracy (macro)** | **Prevalence-weighted one-vs-rest AUC** | **Multiclass log loss** | **Multiclass Brier score** |
| --- | --- | --- | --- | --- | --- | --- | --- |
| Primary frozen model including length of stay | 237 | 215 | 0.793 | 0.732 | 0.854 | 0.617 | 0.323 |
| Exploratory model excluding length of stay | 237 | 215 | 0.776 | 0.564 | 0.797 | 0.643 | 0.336 |

**Panel B.** Class-specific performance of the exploratory model excluding length of stay

| **Outcome class** | **Support, n** | **Precision** | **Recall/sensitivity** | **Specificity** | **F1-score** | **Balanced accuracy** |
| --- | --- | --- | --- | --- | --- | --- |
| Deceased | 17 | 0.385 | 0.294 | 0.964 | 0.333 | 0.629 |
| Ongoing | 36 | NA | 0.000 | 1.000 | NA | 0.500 |
| Recovered | 184 | 0.799 | 0.973 | 0.151 | 0.877 | 0.562 |

AUC, area under the receiver operating characteristic curve; NA, not estimable.

**Supplementary Table S5. Binary deceased-versus-recovered sensitivity analysis of the frozen multiclass classification model.** Ongoing episodes were excluded. Deceased-outcome probabilities from the frozen multiclass model were evaluated without refitting or recalibration. The AUC 95% CI was estimated using 2,000 bootstrap resamples clustered by elephant; calibration measures are presented as point estimates.

**Panel A.** Analysis cohort and discrimination

| **Hospitalization episodes, n** | **Deceased, n (%)** | **Recovered, n (%)** | **AUC (95% CI)** |
| --- | --- | --- | --- |
| 201 | 17 (8.5) | 184 (91.5) | 0.726 (0.585 to 0.839) |

**Panel B.** Calibration of deceased-outcome probabilities

| **Brier score** | **Scaled Brier score** | **Calibration-in-the-large** | **Calibration slope** |
| --- | --- | --- | --- |
| 0.107 | −0.387 | −1.393 | 0.659 |

AUC, area under the receiver operating characteristic curve; CI, confidence interval.

**Supplementary Fig S1. Flow of source records into the external validation cohort.**
Across the three hospitals, 243 source records were screened. Four records from Hospital A did not represent independent clinical admissions and were excluded during initial record review. During subsequent record-level verification, one record from Hospital B was excluded because of an unresolved date inconsistency, and one duplicate hospitalization record from Hospital C was removed. The final external validation cohort comprised 237 hospitalization episodes contributed by 215 elephants. Twenty-two elephants contributed repeated admissions, accounting for 44 episodes.

**Supplementary Fig S2. Binary deceased-versus-recovered sensitivity analysis.**

(A) Receiver operating characteristic curve for discrimination between deceased and recovered hospitalization outcomes. (B) Calibration plot comparing mean predicted probabilities of the deceased outcome with observed event rates. Ongoing episodes were excluded, and the deceased-outcome probabilities generated by the frozen multiclass model were evaluated without refitting or recalibration. Error bars indicate exact 95% binomial confidence intervals. The dashed diagonal line indicates no discrimination in panel A and perfect calibration in panel B.
