# Supplementary material for: External validation of a machine learning model for classifying hospitalization outcomes in Asian elephants across three elephant hospitals in Thailand
Source: PLoS One. 2026 Nov 11;21(11):e0362469. doi: 10.1371/journal.pone.0362469 (PMC13754164; doi:10.1371/journal.pone.0362469)
Supplement: Supplementary file 2 [file pone.0362469.s003.docx]

**Response to Reviewer #1**

**Overall assessment:** Potentially publishable and valuable, but the claims need to be narrowed and the reporting made more transparent.

**Overall comment:** This is a worthwhile, clearly written study that addresses an important gap: external validation of a clinical prediction/classification model in a wildlife hospital context. The validation design is appropriate in principle because the authors apply a frozen previously developed random forest model to temporally distinct 2025 records from three elephant hospitals without refitting or recalibration. However, substantial revision is needed to ensure the language, claims and performance interpretation match what the model can actually support.
**Response:** We sincerely thank the reviewer for the careful, constructive, and detailed evaluation of our manuscript and for recognizing the potential value of this study. In response, we have substantially revised the manuscript to align the interpretation and claims with the information available to the model. Specifically, we have reframed the model as a hospitalization outcome classification model rather than an admission-time prognostic tool; clarified that the study comprised temporal validation at the original development center together with institutional validation at two additional hospitals; improved transparency regarding cohort construction, exclusions, and repeated admissions; benchmarked model accuracy against a recovered-only majority-class classifier; expanded class-specific and quantitative calibration reporting; and added sensitivity analyses addressing repeated admissions and the inclusion of length of stay. We have also revised the Discussion and Conclusion to provide a more cautious interpretation of mortality classification, calibration, and potential clinical application. All revisions are described point by point below.

**Highest-priority issues**
**Point 1:** Main interpretive concern: Length of stay is included as a predictor, so the model should not be framed as an admission-time prognostic tool.
Priority 1. Reframe the model: Because length of stay is included as a predictor, avoid presenting the model as an admission-time prognostic tool.
**Response 1:** We thank the reviewer for identifying this important interpretive issue. We fully agree that length of stay is generated during hospitalization and is not available at admission. Moreover, this variable may reflect not only the patient’s clinical course but also discharge practices, inpatient capacity, follow-up arrangements, and the administrative timing of outcome ascertainment. Accordingly, the validated model should not be interpreted as an admission-time prognostic tool.

Length of stay was retained in the primary analysis because the purpose of the study was to externally validate the previously developed frozen model without altering its original predictor set, preprocessing pipeline, fitted parameters, or class-weighting structure. Removing length of stay from the primary analysis would therefore have resulted in evaluation of a different model rather than strict external validation of the original model.

In direct response to the reviewer’s concern, we additionally fitted a separate exploratory random forest model using the original development dataset but restricted to age, sex, and primary disease category. The exploratory model was then evaluated in the same external validation cohort. Exclusion of length of stay resulted in lower overall discrimination and balanced accuracy, and the exploratory model did not classify any hospitalization episodes as ongoing. These results are reported without interpretive language in the revised Results and Supplementary Table S4B. Their implications are considered in the revised Discussion.

We have revised the manuscript throughout to distinguish hospitalization outcome classification using information accumulated during care from prognosis at admission. The revised Introduction now states (Lines 101–104):

“Because the original model included length of stay, the present validation was interpreted as an evaluation of hospitalization outcome classification using demographic, diagnostic, and hospitalization-duration information accumulated during care, rather than as validation of prognosis at admission.”

We have also added the following clarification to the Methods, Data inclusion and predictor variables section (Lines 168–177):

“Length of stay was generated during hospitalization and was therefore not available at admission. This variable may capture information arising during the clinical course and may also reflect discharge practices, inpatient capacity, follow-up arrangements, ongoing-care definitions, and the administrative timing of outcome ascertainment. For ongoing episodes, length of stay was administratively censored at 31 December 2025 and was therefore additionally linked to the prespecified administrative cutoff used to define outcome status. Accordingly, the primary model incorporates information accumulated during hospitalization and should be interpreted as a hospitalization-course classification model rather than an admission-time prognostic tool. Length of stay was retained in the primary analysis to preserve external validation of the frozen model and its original predictor set.”

The exploratory analysis excluding length of stay is now described in the Methods as follows (Lines 244-249):

“To examine the dependence of classification performance on length of stay, a separate exploratory random forest model was fitted using the original development dataset but restricted to age, sex, and primary disease category. This model was then evaluated in the same external validation cohort. Because the model was fitted separately after removal of a predictor, this analysis was considered exploratory and did not represent external validation of the frozen primary model.”

The corresponding Results now state (Lines 362–364; Supplementary Table S4B):

“In contrast, the exploratory model excluding length of stay showed lower overall discrimination and balanced accuracy and did not classify any episodes as ongoing (Supplementary Table S4B).”

Finally, the interpretation of this finding has been added to the revised Discussion and Limitations (Lines 474–483):

“the frozen model included length of stay, which is generated during hospitalization and may reflect not only the patient’s clinical course but also discharge practices, inpatient capacity, follow-up arrangements, ongoing-care definitions, and the administrative timing of outcome ascertainment. Retaining this variable was necessary to preserve the original predictor set during external validation, but its inclusion limits interpretation of the model as an admission-time prognostic tool. In the exploratory analysis, the model excluding length of stay showed lower overall discrimination and balanced accuracy and did not classify any ongoing episodes. Although this separately fitted model was exploratory, these findings suggest that hospitalization-duration information contributed to classification performance”

**Point 2:** Recovered cases dominate the cohort.

Priority 2. Benchmark against the majority class: Recovered cases are 78.9% of the cohort and overall accuracy is 0.797, so accuracy alone is not impressive without comparison to a recovered-only classifier.

**Response 2:** We thank the reviewer for highlighting the importance of interpreting overall accuracy in the context of the dominant recovered outcome. We agree that accuracy alone may give an overly favorable impression of model performance when one outcome class constitutes the majority of the cohort.

Following final record-level verification of the validation cohort, the outcome distribution was updated to 17 deceased, 36 ongoing, and 184 recovered hospitalization episodes among 237 episodes in total. Recovered episodes therefore represented 77.6% of the final cohort. A classifier assigning every hospitalization episode to the recovered category would achieve an accuracy of 0.776, compared with an overall accuracy of 0.793 for the frozen random forest model, corresponding to an absolute accuracy gain of only 0.017.

In response to the reviewer’s comment, we added the recovered-only majority-class classifier as an explicit benchmark in the performance evaluation. The revised Methods now state (Lines 213–216):

“To contextualize overall classification accuracy in the presence of class imbalance, model accuracy was compared with a majority-class benchmark that classified every hospitalization episode as recovered, the most prevalent outcome in the validation cohort.”

We also revised the Results to avoid interpreting overall accuracy in isolation. The revised Results now state (Lines 302–306):

“Overall accuracy was 0.793, only modestly higher than the recovered-only majority-class benchmark of 0.776, corresponding to an absolute accuracy gain of 0.017. Macro-averaged balanced accuracy was 0.732, and performance differed substantially across outcome classes, with lower precision, recall, and F1-score for deceased episodes than for ongoing and recovered episodes (Table 2).”

The numerical comparison is now reported directly in the NEW Table 2, panel A (Lines 323–335), including the overall model accuracy (0.793), recovered-only majority-class accuracy (0.776), and absolute accuracy gain (0.017).

We have also added this issue explicitly to the Limitations (Lines 456–459):

“recovered episodes constituted the majority of the validation cohort, and overall accuracy was only modestly higher than that of the recovered-only majority-class benchmark. Overall accuracy should therefore be interpreted together with balanced accuracy, class-specific performance, probabilistic error, and calibration rather than as a standalone measure of model performance.”

**Point 3:** Deceased events are sparse

Priority 3. Be cautious about mortality classification: There are only 17 deceased cases; deceased-class performance and calibration will be uncertain.
**Response 3:** We thank the reviewer for emphasizing the limited number of deceased outcomes and the resulting uncertainty in mortality-focused evaluation. We agree that the 17 deceased hospitalization episodes provide a relatively small event count for estimating deceased-class discrimination, classification performance, and calibration, and that these findings should therefore be interpreted cautiously.

We have revised the manuscript to make this limitation explicit throughout the presentation and interpretation of the results. The revised Abstract now states (Lines 33–35):

“The limited number of deceased episodes resulted in substantial uncertainty around mortality-focused performance and calibration estimates.”

In the main Results, we now explicitly emphasize the poorer performance of the deceased class (Lines 304–308):

“Macro-averaged balanced accuracy was 0.732, and performance differed substantially across outcome classes, with lower precision, recall, and F1-score for deceased episodes than for ongoing and recovered episodes (Table 2). Among the 17 deceased episodes, precision was 0.207 (95% CI, 0.069–0.367), recall was 0.353 (95% CI, 0.133–0.588), and the F1-score was 0.261 (95% CI, 0.097–0.421) (Table 2, panel B).”

Class-specific estimates and their 95% confidence intervals are reported in Table 2, panel B (Lines 329–335), with the deceased class comprising 17 hospitalization episodes.

We have also revised the calibration Results to acknowledge the uncertainty associated with the small number of deceased events (Lines 340–342):

“Uncertainty was greatest for the deceased class, consistent with the limited number of deceased episodes.”

Similarly, the binary deceased-versus-recovered sensitivity analysis is now interpreted cautiously (Lines 361–364):

“In the binary deceased-versus-recovered sensitivity analysis, discrimination was moderate, with an AUC of 0.726 (95% CI, 0.585–0.839). The wide confidence interval indicated substantial uncertainty associated with the limited number of deceased episodes (Supplementary Table S5; Supplementary Fig S2).”

The Discussion further emphasizes that deceased-class performance should be interpreted cautiously (Lines 390–402), and this issue is stated explicitly as the first limitation (Lines 453–455):

“First, the number of deceased cases in the external dataset was relatively small, which reduced statistical precision for mortality-focused analyses and widened uncertainty around class-specific performance estimates.”

**Point 4:** The ongoing outcome is not a stable final endpoint.

Priority 4. Define ongoing clearly: Ongoing is a dynamic state and may reflect hospital workflow, mobile follow-up or administrative status rather than a final clinical outcome.
**Response 4:** We thank the reviewer for highlighting the need for a clearer operational definition of the ongoing category. We agree that ongoing should not be interpreted as a stable final clinical endpoint because it represents patient status at a predefined administrative cutoff and may also reflect local care and follow-up practices.

We have therefore revised the Methods to define ongoing explicitly as a status assessed at the administrative cutoff of 31 December 2025, rather than as a final outcome. The revised Methods include the following operational definition (Lines 155–161):

“Clinical outcome status was defined at the administrative cutoff of 31 December 2025. Episodes were classified as: 1) deceased, if death occurred during the hospitalization episode; 2) recovered, if the episode had concluded following clinical recovery; and 3) ongoing, if the episode had not yet been classified as recovered or deceased and the elephant remained under active veterinary management at the administrative cutoff. Ongoing status did not necessarily indicate continued physical hospitalization and could include continued home-based or mobile follow-up according to local hospital practice.”

We also revised the Results to refer explicitly to ongoing episodes at the administrative cutoff (Lines 287–289):

“Outcome distributions differed across hospitals, with ongoing episodes concentrated at Hospital C and relatively few ongoing episodes at Hospitals A and B. Deceased episodes were uncommon overall (Table 1).”

In the Discussion, we now clarify the operational meaning of this category (Lines 413–415):

“Importantly, ongoing status represented active veterinary management at the administrative cutoff and did not necessarily indicate continued physical hospitalization; in some settings, it also included home-based or mobile follow-up.”

Finally, we strengthened the corresponding limitation (Lines 465–468):

“Fourth, the ongoing category reflects a dynamic clinical and administrative status rather than a final endpoint, and its interpretation may differ across hospitals depending on workflow, monitoring practices, follow-up arrangements, and discharge timing.”

**Point 5:** Priority 5. Clarify external validation type: One hospital is the original development centre, so this is temporal validation plus partial institutional validation, not a fully independent multi-centre validation.
**Response 5:** We thank the reviewer for this important clarification. We agree that the validation design should not be described in a way that implies that all three participating hospitals were institutionally independent of the original model-development setting. Hospital C was the original development center, although the 2025 hospitalization episodes used in the present study were temporally distinct from the 2019–2024 development cohort. Hospitals A and B were additional institutions that were not represented in the development dataset.

We have therefore revised the manuscript to describe the study explicitly as temporal validation at the original development center together with institutional validation at two additional hospitals.

The revised Abstract now states (Lines 22–26):

“This study evaluated a previously developed random forest model for classifying hospitalization outcomes in Asian elephants using temporally distinct 2025 data from three elephant hospitals in Thailand. The validation comprised temporal validation at the original development center and institutional validation at two additional hospitals.”

We also revised the study objective in the Introduction (Lines 98–104):

“The objective of this study was to evaluate that previously developed model under both temporal and institutional variation. The validation comprised temporal validation at the original development center using 2025 data and institutional validation at two additional elephant hospitals.”

The validation structure is now stated explicitly in the Methods, Study design and data sources section (Lines 122–128):

“Accordingly, the validation design comprised temporal validation at the original development center (Hospital C) together with institutional validation at Hospitals A and B, which were not represented in the development cohort.”

We have also retained an explicit limitation in the Discussion (Lines 470–474):

“Accordingly, this study represents external validation under temporal shift and partial institutional variation rather than a fully independent multi-institutional validation in which all centers are entirely external to model development.”

Finally, the Conclusion has been revised to maintain the same distinction between temporal and institutional validation (Lines 494–496):

“This study evaluated a previously developed frozen random forest model for classifying hospitalization outcomes in Asian elephants through temporal validation at the original development center and institutional validation at two additional elephant hospitals in Thailand.”

**Point 6:** Priority 6. Report exclusions and repeated admissions: The authors should provide a flow summary and clarify whether the 237 admissions represent unique elephants.
**Response 6:** We thank the reviewer for highlighting the need for greater transparency regarding cohort construction and repeated admissions. We have revised the manuscript to provide a complete record flow and to clarify that the 237 hospitalization episodes did not represent 237 unique elephants.

Across the three hospitals, 243 source records were screened. Four records did not represent independent clinical admissions and were excluded during initial record review. During record-level verification, one record was excluded because of an unresolved date inconsistency and one duplicate hospitalization record was removed. The final analytic cohort therefore comprised 237 hospitalization episodes contributed by 215 unique elephants. Twenty-two elephants contributed repeated admissions, accounting for 44 hospitalization episodes.

We have clarified the unit of analysis and handling of repeated admissions in the Methods (Lines 188–191):

“The unit of analysis was the hospitalization episode. Elephants with more than one eligible hospitalization during the study period could therefore contribute multiple episodes to the primary analysis. Repeated admissions by the same elephant were retained because each represented a separate hospitalization episode.”

We also revised the bootstrap procedure to account for repeated episodes from the same elephant (Lines 232–234):

“Ninety-five percent confidence intervals were estimated using 2,000 bootstrap resamples clustered by elephant to account for repeated hospitalization episodes contributed by the same individual.”

In addition, we added a first-episode-only sensitivity analysis (Lines 235–238):

“To assess the influence of repeated admissions on the primary findings, a sensitivity analysis was performed after retaining only the first eligible hospitalization episode for each elephant. Model performance in this first-admission-only cohort was compared with that of the complete episode-level cohort.”

The revised Results now provide the complete cohort flow (Lines 275–283):

“Across the three hospitals, 243 source records were screened, including 33 from Hospital A, 84 from Hospital B, and 126 from Hospital C. Four source records from Hospital A were excluded because they did not represent independent clinical admissions. During subsequent record-level verification, one record from Hospital B was excluded because of an unresolved date inconsistency, and one duplicate hospitalization record from Hospital C was removed. The final external validation cohort therefore comprised 237 hospitalization episodes, including 29 from Hospital A, 83 from Hospital B, and 125 from Hospital C, contributed by 215 elephants. Twenty-two elephants contributed repeated admissions, accounting for 44 episodes (Supplementary Figure S1).”

The first-admission-only sensitivity analysis is reported in the Results (Lines 357–359):

“Restricting the analysis to the first eligible hospitalization episode per elephant produced performance estimates similar to those of the complete episode-level cohort (Supplementary Table S4A).”

A detailed record-flow diagram has also been added as Supplementary Fig S1.

Finally, we added repeated admissions explicitly to the Limitations (currently Lines 460–465):

“Third, twenty-two elephants contributed more than one eligible hospitalization episode, accounting for 44 episodes, so episode-level observations were not completely independent. To account for this clustering, confidence intervals were estimated using bootstrap resampling clustered by elephant, and a sensitivity analysis retaining only the first eligible hospitalization episode per elephant produced similar overall performance estimates. Nevertheless, some within-elephant dependence may remain in the episode-level analysis.”

**Point 7:** Priority 7. Strengthen calibration reporting: Visual decile plots are useful but insufficient, especially with sparse death events.
**Response 7:** We thank the reviewer for this important suggestion. We agree that graphical calibration based on probability deciles alone is insufficient to characterize probabilistic performance, particularly for the deceased class, which contained only 17 events.

We have therefore expanded the calibration assessment to include quantitative measures in addition to graphical calibration. Calibration was assessed separately for each outcome class using the Brier score, scaled Brier score, calibration-in-the-large, and calibration slope, while overall probabilistic performance was additionally summarized using the multiclass Brier score. Ninety-five percent confidence intervals for these quantitative measures were estimated using 2,000 bootstrap resamples clustered by elephant. We also added exact 95% binomial confidence intervals to the observed event rates displayed in the calibration plots.

The revised Methods now state (Lines 218–226):

“Calibration was assessed separately for each outcome class using a one-vs-rest framework. Graphical calibration was evaluated by comparing mean predicted probabilities with observed event rates across deciles of predicted probability, with exact 95% binomial confidence intervals for the observed event rates. Quantitative calibration was summarized using the Brier score, scaled Brier score, calibration-in-the-large, and calibration slope. Overall probabilistic performance was additionally summarized using the multiclass Brier score. Ninety-five percent confidence intervals for the quantitative calibration measures and multiclass Brier score were estimated using 2,000 bootstrap resamples clustered by elephant. No post hoc recalibration was performed.”

We also expanded the calibration Results (Lines 336–342):

“Calibration patterns differed across outcome classes. Calibration-in-the-large was −1.594 (95% CI, −2.325 to −1.081) for deceased, −0.195 (95% CI, −0.673 to 0.212) for ongoing, and 1.131 (95% CI, 0.810 to 1.500) for recovered outcomes (Figure 3; Supplementary Table S2). Calibration slopes were 0.635 (95% CI, 0.239–1.042), 0.864 (95% CI, 0.571–1.280), and 1.164 (95% CI, 0.873–1.594), respectively. Uncertainty was greatest for the deceased class, consistent with the limited number of deceased episodes. The overall multiclass Brier score was 0.323 (95% CI, 0.271–0.377).”

The complete quantitative calibration results, including class-specific Brier scores and scaled Brier scores with 95% confidence intervals, are provided in Supplementary Table S2. Figure 3 was also revised to display exact 95% binomial confidence intervals around the observed event rates within each probability decile (Lines 344–348).

**Section comments and suggested edits**

**1. Title and short title**
**Point 8:** Title and short title
The current title is broadly accurate, but it may imply a general clinical prediction or admission-time prognosis model. Because length of stay is included, the title should make clear that this is hospitalization outcome classification.
 The title should clarify that the model classifies hospitalization outcomes rather than predicting prognosis at admission. This distinction matters because one of the predictors is length of stay, which is not known at admission and may be affected by the clinical course and hospital workflow.
**Response 8:** We thank the reviewer for this helpful suggestion. We agree that the title should more explicitly distinguish hospitalization outcome classification from prognosis at the time of admission, particularly because length of stay is included among the predictors.

We have therefore revised the title (Lines 1–2) from:

“External validation of a machine learning model for clinical outcome classification in hospitalized Asian elephants across three elephant hospitals in Thailand”

to:

**“External validation of a machine learning model for classifying hospitalization outcomes in Asian elephants across three elephant hospitals in Thailand.”**

We have also revised the short title to:

**“Hospitalization outcome classification in Asian elephants.”**

This terminology is consistent with the revised framing throughout the manuscript, in which the model is interpreted as a hospitalization-course classification model rather than an admission-time prognostic tool.

**2. Abstract**

**Point 9:** The abstract should provide more quantitative information. Phrases such as “retained useful overall performance” and “acceptable agreement” are difficult to interpret without the sample size, outcome distribution and performance metrics. Please report N = 237 admissions, the class distribution, the main multiclass metrics, and the binary sensitivity analysis AUC. The abstract should also state that performance was weaker for deceased cases and that mortality-related conclusions are limited by sparse death events.
 Add to abstract: N = 237 admissions; deceased = 17, ongoing = 33, recovered = 187; accuracy = 0.797; macro/balanced accuracy = 0.741; multiclass AUC = 0.862; log loss = 0.564; binary deceased-versus-recovered AUC = 0.722, 95% CI 0.594–0.834.
**Response 9:** We thank the reviewer for this helpful suggestion. We agree that the Abstract should provide quantitative performance measures rather than rely on qualitative descriptions such as “retained useful overall performance” or “acceptable agreement.” We have therefore substantially revised the Abstract to report the size and composition of the validation cohort, the majority-class benchmark, the principal multiclass performance measures, the binary deceased-versus-recovered sensitivity analysis, and the uncertainty associated with the limited number of deceased episodes.

During revision, final record-level verification of the validation cohort resulted in updates to several outcome classifications while the total cohort size remained unchanged at 237 hospitalization episodes. The final verified cohort comprised 17 deceased, 36 ongoing, and 184 recovered episodes contributed by 215 elephants. All performance metrics were recalculated using this verified cohort. Accordingly, the final estimates differ slightly from those reported in the original submission and quoted in the reviewer’s comment.

The revised Abstract now states (Lines 28–38):

“The final validation cohort comprised 237 hospitalization episodes contributed by 215 elephants, including 17 deceased, 36 ongoing, and 184 recovered episodes. Overall accuracy was 0.793, compared with 0.776 for a recovered-only majority-class classifier. Macro-averaged balanced accuracy was 0.732, the prevalence-weighted one-vs-rest area under the receiver operating characteristic curve (AUC) was 0.854, and multiclass log loss was 0.617. Classification performance was stronger for ongoing and recovered episodes than for deceased episodes. The limited number of deceased episodes resulted in substantial uncertainty around mortality-focused performance and calibration estimates. Calibration patterns differed across outcome classes, with an overall multiclass Brier score of 0.323. In the binary deceased-versus-recovered sensitivity analysis, the AUC was 0.726 (95% confidence interval, 0.585–0.839), indicating moderate but imprecise discrimination.”

We also removed the previous qualitative statements describing the model as having “useful overall performance” or “acceptable agreement” and replaced them with metric-based reporting and a more cautious concluding interpretation.

**3. Introduction**

**Point 10:** The introduction provides a strong rationale for external validation and explains why elephant hospital data may differ by institution, time period, case mix and clinical workflow. The introduction uses prognostic language quite broadly. That is understandable, but this particular model includes length of stay, so the manuscript needs to distinguish admission-time prognosis from outcome classification using information accumulated during hospitalization.
 Please distinguish between admission-time prognosis and outcome classification using hospital-course information. This is important because the original model includes length of stay, which is not available at admission and may be influenced by the outcome and hospital workflow.
 Suggested wording- Because the original model included length of stay, the present validation should be interpreted as an evaluation of hospitalization outcome classification using routinely recorded demographic, diagnostic and hospitalization-duration variables, rather than as validation of an admission-time prognostic tool.
 The objective should also acknowledge that one validation hospital was the original development centre.
**Response 10:** We thank the reviewer for this important distinction. We agree that the Introduction should clearly separate the broader literature on prognostic modeling from the specific interpretation of the model evaluated in the present study. Because length of stay is generated during hospitalization and is not available at admission, we have revised the Introduction to avoid framing the present model as an admission-time prognostic tool.

We first revised the general framing in the opening paragraph by replacing “prognostic assessment” with “clinical outcome assessment” when referring specifically to elephant hospital care (Lines 51–53):

“As a result, clinical outcome assessment in elephant hospital settings has implications not only for clinical care, but also for animal welfare, allocation of limited medical resources, and decisions regarding prolonged treatment or end-of-life management.”

We retained prognostic terminology where it refers to the broader veterinary and wildlife literature, but revised the study-specific objective to distinguish clearly between prognosis at admission and classification using information accumulated during hospitalization. The revised objective now states (Lines 98–104):

“The objective of this study was to evaluate that previously developed model under both temporal and institutional variation. The validation comprised temporal validation at the original development center using 2025 data and institutional validation at two additional elephant hospitals. Because the original model included length of stay, the present validation was interpreted as an evaluation of hospitalization outcome classification using demographic, diagnostic, and hospitalization-duration information accumulated during care, rather than as validation of prognosis at admission.”

We further clarified that the frozen model was applied without updating or recalibration to hospitalization episodes active during 2025 and added sensitivity analyses addressing repeated admissions, exclusion of length of stay, and binary deceased-versus-recovered classification (Lines 104–110).

**4. Methods: study design and data sources**
**Point 11:** The authors appropriately state that the model was applied without refitting, recalibration or updating. This is important and should remain prominent.
 Because one hospital was the original development centre, this is not a fully independent multi-institutional validation. It is a temporal validation at the original centre plus institutional validation at two additional hospitals.
 Please clarify throughout the manuscript that this is temporal validation with partial institutional external validation, rather than a fully independent validation in which all hospitals were external to the original model-development setting.
**Response 11:** We thank the reviewer for this important clarification. We agree that the validation design should distinguish temporal validation at the original development center from institutional validation at hospitals that were not represented during model development. We have retained the fact that the frozen model was applied without refitting, recalibration, or updating as a prominent feature of the study design.

The revised Methods now state (Lines 122–128):

“Although one hospital in the validation cohort corresponded to the original development center (Hospital C), no records from the development period were reused. All hospitalization episodes included in the external validation dataset were temporally distinct from the development cohort and were not used at any stage of model development or internal validation. Accordingly, the validation design comprised temporal validation at the original development center (Hospital C) together with institutional validation at Hospitals A and B, which were not represented in the development cohort.”

We also retained the following statement at the end of the Study design and data sources section (Lines 145-147):

“The aim of the external validation was to assess model performance under temporal and institutional variation by applying the frozen outcome classification model to eligible hospitalization episodes without model refitting, recalibration, or updating.”

This distinction has also been harmonized across the Abstract and Introduction, which now describe the study as temporal validation at the original development center together with institutional validation at two additional hospitals. In the Limitations, we further clarify that the study represents external validation under temporal shift and partial institutional variation rather than a fully independent multi-institutional validation in which all participating centers were external to model development.

**5. Methods: inclusion criteria and exclusions**
**Point 12:** The manuscript states which records were eligible but does not report how many 2025 records were screened or excluded. This is a key transparency issue because exclusions could bias validation performance.
 Please provide a record flow summary showing the number of admissions screened at each hospital, the number excluded, and reasons for exclusion. This could be included in the Results or as a supplementary flow diagram.
 Information needed: Total 2025 admissions available by hospital; number excluded for missing age, sex, disease category, outcome, admission date or discharge date; and the final analytic dataset by hospital.
 Suggested wording - A total of X admissions were screened across the three hospitals. Of these, X were excluded because of missing or unclear data, including X with missing age, X with missing disease category, X with missing outcome status, and X with unclear admission or discharge dates. The final analytic dataset included 237 admissions.
**Response 12:** We thank the reviewer for highlighting this important transparency issue. We conducted a record-level audit of the source data and have revised the manuscript to report the number of source records screened, exclusions and their reasons, and the final analytic cohort both overall and by hospital.

Across the three hospitals, 243 source records were screened: 33 from Hospital A, 84 from Hospital B, and 126 from Hospital C. Four source records from Hospital A were excluded because they did not represent independent clinical admissions. During subsequent record-level verification, one record from Hospital B was excluded because of an unresolved date inconsistency, and one duplicate hospitalization record from Hospital C was removed. The final analytic cohort therefore comprised 237 hospitalization episodes: 29 from Hospital A, 83 from Hospital B, and 125 from Hospital C.

No additional otherwise eligible hospitalization episodes were excluded solely because of missing age, sex, primary disease category, outcome status, or admission date. A missing discharge date was not itself an exclusion criterion for episodes classified as ongoing because length of stay for these episodes was administratively censored at 31 December 2025.

The revised Results now state (Lines 275–283):

“Across the three hospitals, 243 source records were screened, including 33 from Hospital A, 84 from Hospital B, and 126 from Hospital C. Four source records from Hospital A were excluded because they did not represent independent clinical admissions. During subsequent record-level verification, one record from Hospital B was excluded because of an unresolved date inconsistency, and one duplicate hospitalization record from Hospital C was removed. The final external validation cohort therefore comprised 237 hospitalization episodes, including 29 from Hospital A, 83 from Hospital B, and 125 from Hospital C, contributed by 215 elephants. Twenty-two elephants contributed repeated admissions, accounting for 44 episodes (Supplementary Fig S1).”

We also added a detailed record-flow diagram as Supplementary Fig S1 showing the number of source records screened and retained at each hospital, together with the reasons for exclusion. The figure confirms the progression from 243 screened source records to 237 hospitalization episodes in the final external validation cohort.

**6. Methods: outcome definitions**
**Point 13:** The “ongoing” outcome is central to the model but is not a stable final endpoint. It needs clearer operational definition. The Discussion notes that ongoing status may include home-based or mobile follow-up, but this belongs in the Methods as well.
 Please define the ongoing category more clearly. It is currently unclear whether ongoing consistently means physically hospitalized, still under active veterinary management, receiving mobile follow-up, or not yet administratively discharged. Because this category may reflect hospital workflow as much as clinical status, this definition is essential for interpreting the model.
 Also needed - The exact data extraction date, whether ongoing status was determined consistently across hospitals, and whether ongoing cases were later resolved after the extraction date.
**Response 13:** We thank the reviewer for highlighting the need for a more explicit operational definition of the ongoing category. We agree that ongoing represents a clinical and administrative status at a predefined cutoff rather than a stable final clinical endpoint and that it may reflect both active veterinary management and local follow-up practices.

We have therefore revised the Methods to distinguish clearly between the administrative cutoff used to assign outcome status and the subsequent date of data extraction. Outcome status was anchored to 31 December 2025, whereas the validation dataset was extracted on 29 January 2026. During manual record verification, outcome status was reviewed using the same operational definition across all three hospitals.

The revised Methods now state (Lines 155–167):

“Clinical outcome status was defined at the administrative cutoff of 31 December 2025. Episodes were classified as: 1) deceased, if death occurred during the hospitalization episode; 2) recovered, if the episode had concluded following clinical recovery; and 3) ongoing, if the episode had not yet been classified as recovered or deceased and the elephant remained under active veterinary management at the administrative cutoff. Ongoing status did not necessarily indicate continued physical hospitalization and could include continued home-based or mobile follow-up according to local hospital practice. The validation dataset was extracted on 29 January 2026. During manual record verification, outcome status was reviewed using the same operational definition across all three hospitals with reference to the 31 December 2025 administrative cutoff. For ongoing episodes, length of stay was administratively censored at 31 December 2025. Subsequent clinical resolution after the administrative cutoff was not used to relabel outcome status in the validation dataset. Clinical outcomes occurring after the data extraction date were not systematically ascertained.”

Some episodes classified as ongoing at the administrative cutoff were subsequently known to have recovered after 31 December 2025. These later outcomes were not used to change the outcome labels used for external validation because the analysis was defined according to clinical status at the prespecified administrative cutoff. Clinical outcomes occurring after the data extraction date of 29 January 2026 were not systematically ascertained for the present validation analysis.

We have also revised the Discussion and Limitations to emphasize that the ongoing category represents a dynamic clinical and administrative state rather than a final endpoint and that its interpretation may be influenced by hospital workflow, monitoring practices, follow-up arrangements, and discharge timing.

**7. Methods: predictor variables**
**Point 14:** Length of stay is included as a predictor. This is the most important methodological and interpretive issue in the manuscript.
 Length of stay is not available at admission and may be directly affected by outcome, discharge policy, case severity, hospital capacity, follow-up practices and data extraction timing. For ongoing cases, length of stay is censored at 31 December 2025, making it particularly tied to outcome definition.
 The inclusion of length of stay should be more explicitly discussed as a potential source of outcome-related information. Because length of stay is determined during hospitalization and may be affected by discharge practices, clinical deterioration, ongoing care definitions and data extraction timing, the model should not be described as an admission-time prognostic tool. Please revise the manuscript accordingly and consider an additional sensitivity analysis using only predictors available at admission.
 If possible, evaluate or develop a model using only age, sex and primary disease category. Even if performance decreases, it would help distinguish true admission-time prognostic performance from hospital-course classification.
 Suggested wording - Because length of stay was included as a predictor, the model incorporates information generated during hospitalization and should not be interpreted as an admission-time prognostic tool. Length of stay may also reflect hospital workflow, discharge policies and follow-up practices, in addition to patient condition.
**Response 14:** We thank the reviewer for emphasizing this important methodological and interpretive issue. We agree that length of stay is generated during hospitalization and is not available at admission. It may contain information arising during the clinical course while also reflecting hospital-specific factors such as discharge practices, inpatient capacity, follow-up arrangements, ongoing-care definitions, and the administrative timing of outcome ascertainment. In addition, length of stay for ongoing episodes was administratively censored at 31 December 2025, linking this predictor to the prespecified administrative cutoff used to define outcome status.

We retained length of stay in the primary analysis because the objective was to externally validate the previously developed frozen model using its original predictor set. Removing this predictor from the primary model would constitute evaluation of a different model rather than strict external validation of the original model. We have therefore revised the manuscript throughout to describe the primary model as a hospitalization-course classification model rather than an admission-time prognostic tool.

The revised Methods now state (Lines 168–177):

“Length of stay was generated during hospitalization and was therefore not available at admission. This variable may capture information arising during the clinical course and may also reflect discharge practices, inpatient capacity, follow-up arrangements, ongoing-care definitions, and the administrative timing of outcome ascertainment. For ongoing episodes, length of stay was administratively censored at 31 December 2025 and was therefore additionally linked to the prespecified administrative cutoff used to define outcome status. Accordingly, the primary model incorporates information accumulated during hospitalization and should be interpreted as a hospitalization-course classification model rather than an admission-time prognostic tool. Length of stay was retained in the primary analysis to preserve external validation of the frozen model and its original predictor set.”

In direct response to the reviewer’s suggestion, we also fitted a separate exploratory random forest model using the original development dataset but restricting the predictor set to age, sex, and primary disease category. This model was then evaluated in the same external validation cohort. We emphasize that this was a separately fitted exploratory model rather than the frozen primary model with length of stay simply removed. The revised Methods state (Lines 244–249):

“To examine the dependence of classification performance on length of stay, a separate exploratory random forest model was fitted using the original development dataset but restricted to age, sex, and primary disease category. This model was then evaluated in the same external validation cohort. Because the model was fitted separately after removal of a predictor, this analysis was considered exploratory and did not represent external validation of the frozen primary model.”

The exploratory model without length of stay showed lower performance than the primary frozen model. Overall accuracy was 0.776 compared with 0.793 for the primary model, macro-averaged balanced accuracy was 0.564 compared with 0.732, and the prevalence-weighted one-vs-rest AUC was 0.797 compared with 0.854. Multiclass log loss was 0.643 compared with 0.617, and the multiclass Brier score was 0.336 compared with 0.323. The exploratory model also did not classify any hospitalization episodes as ongoing. These results are reported in Supplementary Table S4B, with the main Results stating (Lines 359–361):

“In contrast, the exploratory model excluding length of stay showed lower overall discrimination and balanced accuracy and did not classify any episodes as ongoing (Supplementary Table S4B).”

We have also incorporated this issue into the Discussion and Limitations (Lines 473–483), emphasizing that retention of length of stay was necessary to preserve the original model specification during external validation but limits interpretation of the model as an admission-time prognostic tool. The reduced performance of the separately fitted exploratory model without length of stay further indicates that hospitalization-duration information contributed to classification performance.

**8. Methods: preprocessing and model application**
**Point 15:** The manuscript says categorical predictors were one-hot encoded and zero-variance predictors were removed. It should be explicit whether this was done using the frozen preprocessing recipe from the development dataset or recalculated in the validation data.
 Please clarify whether zero-variance removal was part of the frozen preprocessing recipe derived from the development dataset, or whether it was recalculated in the external dataset. For strict external validation, factor levels, dummy-variable structure and preprocessing steps should be defined from the development data and applied unchanged to the validation data.
**Response 15:** We thank the reviewer for identifying this point. We agree that the manuscript should make clear that preprocessing was defined from the development data and was not recalculated or adapted using the external validation cohort.

The external validation data were processed using the frozen preprocessing recipe derived during model development. Accordingly, factor levels, the corresponding one-hot encoded dummy-variable structure, and zero-variance predictor removal were determined by the development preprocessing pipeline and applied unchanged to the external validation data. Zero-variance screening was not recalculated using the external validation dataset, and no additional feature engineering, scaling, imputation, or other data-driven preprocessing was introduced during external validation.

We have revised the Methods accordingly (Lines 193–199):

“All preprocessing procedures used during model development were defined by the frozen preprocessing recipe derived from the development dataset and applied unchanged to the external validation data. Factor levels and the corresponding one-hot encoded dummy-variable structure were therefore determined from the development data. Zero-variance predictor removal was also part of the development preprocessing recipe and was not recalculated using the external validation dataset. No additional feature engineering, scaling, imputation, or data-driven preprocessing was introduced during external validation.”

We also retained the statement that the final fitted random forest model, including its hyperparameter settings and class-weighting structure, was loaded from the stored model object and applied directly without refitting, recalibration, or updating (Lines 200–205).

**9. Methods: performance evaluation**
**Point 16:** Overall accuracy may be misleading because recovered cases dominate the cohort. A model that predicted all cases as recovered would already achieve approximately 78.9% accuracy, while the reported accuracy is 79.7%.
 Because recovered cases comprised 78.9% of the validation cohort, overall accuracy should be interpreted against the no-information rate or majority-class classifier. Please report the majority-class baseline accuracy and emphasize balanced accuracy, class-specific recall/precision, log loss and calibration rather than accuracy alone.
**Response 16:** We thank the reviewer for emphasizing this important issue. We agree that overall accuracy can be misleading in an imbalanced multiclass setting and should be interpreted relative to the majority-class benchmark and together with class-balanced, class-specific, and probabilistic performance measures.

Following final record-level verification of the validation cohort, 184 of 237 hospitalization episodes (77.6%) were classified as recovered. A classifier assigning every episode to the recovered category would therefore achieve an accuracy of 0.776, compared with 0.793 for the frozen model, corresponding to an absolute accuracy gain of only 0.017.

We have added the recovered-only majority-class benchmark explicitly to the Methods (Lines 213–216):

“To contextualize overall classification accuracy in the presence of class imbalance, model accuracy was compared with a majority-class benchmark that classified every hospitalization episode as recovered, the most prevalent outcome in the validation cohort.”

We also revised the Results to place overall accuracy in this context (Lines 302–306):

“Overall accuracy was 0.793, only modestly higher than the recovered-only majority-class benchmark of 0.776, corresponding to an absolute accuracy gain of 0.017. Macro-averaged balanced accuracy was 0.732, and performance differed substantially across outcome classes, with lower precision, recall, and F1-score for deceased episodes than for ongoing and recovered episodes (Table 2).”

Table 2 (Lines 323–335) now reports the majority-class benchmark together with macro-averaged balanced accuracy, class-specific performance measures, multiclass discrimination, and probabilistic performance metrics. Quantitative calibration measures with confidence intervals are reported separately in Supplementary Table S2.

Finally, we revised the Limitations (Lines 456–459) to emphasize that overall accuracy was only modestly higher than the recovered-only majority-class benchmark and should not be interpreted as a standalone measure of model performance.

**Point 17:** The phrase “macro-weighted multiclass AUC” is ambiguous. Macro-averaged and weighted-averaged metrics are different.
 Please clarify whether the reported multiclass AUC was macro-averaged, weighted macro-averaged, or calculated using another multiclass method. The current phrase “macro-weighted” is ambiguous.
**Response 17:** We thank the reviewer for identifying this ambiguity. We agree that the term “macro-weighted multiclass AUC” was imprecise and could be interpreted as conflating macro-averaged and prevalence-weighted averaging.

The reported overall AUC was calculated using a one-vs-rest approach, in which an AUC was first calculated separately for each of the three outcome classes and the resulting class-specific AUCs were then averaged using the observed prevalence of each outcome class in the validation cohort as the weight. It is therefore a prevalence-weighted one-vs-rest AUC, not a macro-averaged AUC.

We have revised the terminology throughout the manuscript accordingly. The revised Methods now state (Lines 207–212):

“Overall multiclass performance was summarized using classification accuracy, macro-averaged balanced accuracy, prevalence-weighted one-vs-rest area under the receiver operating characteristic curve (AUC), and multiclass log loss. The prevalence-weighted one-vs-rest AUC was calculated as the weighted mean of the three class-specific AUCs, using the observed prevalence of each outcome class as the weight.”

The same terminology is now used consistently in the Abstract, Results, Table 2, and supplementary analyses. The term “macro-weighted multiclass AUC” has been removed throughout the manuscript.

**Point 18:** Calibration is currently assessed visually using deciles. This is useful, but quantitative calibration metrics would strengthen the paper.
 Calibration assessment would be strengthened by reporting quantitative calibration metrics, such as Brier score, calibration-in-the-large, calibration slope or expected calibration error, with confidence intervals where feasible. If these cannot be estimated reliably because of sparse events, this should be stated.
**Response 18:** We thank the reviewer for this suggestion. We agree that graphical calibration alone is insufficient and have expanded the calibration assessment to include quantitative measures with confidence intervals.

The revised Methods now state (Lines 218–226):

“Calibration was assessed separately for each outcome class using a one-vs-rest framework. Graphical calibration was evaluated by comparing mean predicted probabilities with observed event rates across deciles of predicted probability, with exact 95% binomial confidence intervals for the observed event rates. Quantitative calibration was summarized using the Brier score, scaled Brier score, calibration-in-the-large, and calibration slope. Overall probabilistic performance was additionally summarized using the multiclass Brier score. Ninety-five percent confidence intervals for the quantitative calibration measures and multiclass Brier score were estimated using 2,000 bootstrap resamples clustered by elephant. No post hoc recalibration was performed.”

The revised Results now report the principal quantitative calibration findings (Lines 336–342):

“Calibration-in-the-large was −1.594 (95% CI, −2.325 to −1.081) for deceased, −0.195 (95% CI, −0.673 to 0.212) for ongoing, and 1.131 (95% CI, 0.810 to 1.500) for recovered outcomes (Figure 3; Supplementary Table S2). Calibration slopes were 0.635 (95% CI, 0.239–1.042), 0.864 (95% CI, 0.571–1.280), and 1.164 (95% CI, 0.873–1.594), respectively. Uncertainty was greatest for the deceased class, consistent with the limited number of deceased episodes. The overall multiclass Brier score was 0.323 (95% CI, 0.271–0.377).”

Complete quantitative calibration results, including class-specific Brier scores and scaled Brier scores with 95% confidence intervals, are now provided in Supplementary Table S2. For the deceased class, the relatively wide confidence intervals make the uncertainty associated with the limited number of events explicit.

**10. Results: external validation cohort**
**Point 19:** Table 1 is useful and clearly shows important differences in age, length of stay, disease distribution and outcomes across hospitals.
 The Results should make the implications of the outcome distribution more explicit. Hospital A had no ongoing cases, Hospital C contributed most ongoing cases, and deceased events were sparse overall.
 Please more explicitly describe how outcome distribution differed across hospitals. The absence of ongoing cases in Hospital A and the concentration of ongoing cases in Hospital C are important because they affect hospital-specific metrics and interpretation of transportability.
**Response 19:** We thank the reviewer for highlighting the importance of describing the between-hospital outcome distributions more explicitly. We agree that these differences are important for interpreting hospital-specific performance and the transportability of the model.

During final record-level verification, three hospitalization episodes at Hospital A were confirmed to meet the same operational definition of ongoing status applied across all three hospitals at the administrative cutoff of 31 December 2025. Accordingly, the final verified outcome distribution differed from that reported in the original submission. Hospital A included 2 deceased, 3 ongoing, and 24 recovered episodes; Hospital B included 4 deceased, 3 ongoing, and 76 recovered episodes; and Hospital C included 11 deceased, 30 ongoing, and 84 recovered episodes. Thus, Hospital A no longer had an absent ongoing class, although ongoing episodes remained concentrated at Hospital C.

To avoid duplicating the detailed counts already presented in Table 1, we revised the main Results to emphasize the overall pattern (Lines 287–289):

“Outcome distributions differed across hospitals, with ongoing episodes concentrated at Hospital C and relatively few ongoing episodes at Hospitals A and B. Deceased episodes were uncommon overall (Table 1).”

The updated outcome distributions are reported in Table 1 and Supplementary Table S1. Because ongoing episodes were present at Hospital A in the final verified cohort, hospital-specific balanced accuracy was estimable for all three hospitals in the revised analysis.

**11. Results: multiclass performance**
**Point 20:** The main text gives overall metrics but leaves important class-specific metrics in the supplementary material. Deceased-class recall, precision and F1 score should be in the main Results because mortality classification is clinically important.
 Please include key class-specific metrics in the main Results text, particularly recall, precision and F1 score for the deceased class. Readers should not need to consult supplementary material to evaluate whether the model identifies deceased cases adequately.
 Suggested wording. - Although overall discrimination was acceptable, deceased-class performance was lower than recovered and ongoing performance, with reduced sensitivity and precision. This indicates that the model was less reliable for identifying mortality outcomes than for classifying recovered or ongoing cases.
When reporting accuracy, explicitly compare it with the recovered-only baseline. This is one of the most important interpretive points in the review.
**Response 20:** We thank the reviewer for this important suggestion. We agree that the performance of the deceased class should be visible in the main Results rather than requiring readers to rely on supplementary material, particularly given the clinical importance of mortality classification.

We have therefore retained the comparison of overall accuracy with the recovered-only majority-class benchmark and added the key deceased-class performance measures directly to the main Results. The revised Results now state (Lines 302–308):

“Overall accuracy was 0.793, only modestly higher than the recovered-only majority-class benchmark of 0.776, corresponding to an absolute accuracy gain of 0.017. Macro-averaged balanced accuracy was 0.732, and performance differed substantially across outcome classes, with lower precision, recall, and F1-score for deceased episodes than for ongoing and recovered episodes (Table 2). Among the 17 deceased episodes, precision was 0.207 (95% CI, 0.069–0.367), recall was 0.353 (95% CI, 0.133–0.588), and the F1-score was 0.261 (95% CI, 0.097–0.421) (Table 2, panel B).”

The complete class-specific results for deceased, ongoing, and recovered outcomes, including balanced accuracy and 95% confidence intervals, are presented in Table 2, panel B.

**12. Results: calibration**
**Point 21:** The manuscript says predicted probabilities generally corresponded with observed event rates. This should be more cautious, especially for deceased cases, where there are only 17 events.
 The calibration results should be interpreted more cautiously, especially for deceased cases. With only 17 deaths, decile-based observed mortality rates may be unstable. Please avoid broad statements that calibration was acceptable unless supported by quantitative calibration statistics or confidence intervals.
**Response 21:** We thank the reviewer for this important caution. We agree that broad statements regarding calibration are not appropriate, particularly for the deceased class, for which only 17 events were available and decile-based observed event rates are therefore uncertain.

We have removed the previous general statement that predicted probabilities “generally corresponded with observed event rates” and replaced it with quantitative calibration results and their confidence intervals. The revised Results now state (Lines 336–342):

“Calibration patterns differed across outcome classes. Calibration-in-the-large was −1.594 (95% CI, −2.325 to −1.081) for deceased, −0.195 (95% CI, −0.673 to 0.212) for ongoing, and 1.131 (95% CI, 0.810 to 1.500) for recovered outcomes (Figure 3; Supplementary Table S2). Calibration slopes were 0.635 (95% CI, 0.239–1.042), 0.864 (95% CI, 0.571–1.280), and 1.164 (95% CI, 0.873–1.594), respectively. Uncertainty was greatest for the deceased class, consistent with the limited number of deceased episodes. The overall multiclass Brier score was 0.323 (95% CI, 0.271–0.377).”

In addition, Figure 3 (Lines 344–348) now displays exact 95% binomial confidence intervals around the observed event rates within each probability decile. Complete quantitative calibration results, including class-specific Brier scores, scaled Brier scores, calibration-in-the-large, and calibration slopes with 95% confidence intervals, are provided in Supplementary Table S2.

We therefore avoid characterizing calibration broadly as “acceptable” in the Results and instead report the observed class-specific calibration patterns together with their uncertainty. The interpretation of these findings, particularly the limitations of deceased-class calibration, is addressed more cautiously in the revised Discussion.

**13. Results: disease-stratified performance**
**Point 22:** The disease-stratified analyses are useful descriptively, but some disease categories have small numbers or lack one or more outcome classes.
 The disease-stratified analyses should remain clearly labelled as descriptive and exploratory. The authors should avoid implying that the model is more or less valid for particular disease categories unless there are sufficient numbers of all outcome classes.
**Response 22:** We thank the reviewer for this important clarification. We agree that the disease-stratified analyses should be interpreted only as descriptive and exploratory because several disease categories contained small numbers of hospitalization episodes and, in some subgroups, one or more outcome classes were absent.

We have therefore revised the Methods to make the purpose and limitations of these analyses explicit (Lines 239–243):

“Descriptive performance summaries were additionally generated by disease category for subgroups with at least 15 hospitalization episodes. Balanced accuracy and the prevalence-weighted one-vs-rest AUC could not be estimated for certain disease categories because one or more outcome classes were absent within those subgroups. These analyses were conducted to characterize patterns of model behavior rather than to support formal statistical inference.”

We also revised the Results to avoid implying disease-specific model validity (Lines 351–355):

“Exploratory performance stratified by primary disease category is presented in Supplementary Table S3 for disease groups containing at least 15 hospitalization episodes. Performance estimates varied across disease categories, but several subgroups contained few or no observations for one or more outcome classes. These analyses were considered descriptive and exploratory and were not used to infer disease-specific model validity.”

Supplementary Table S3 is likewise explicitly presented as exploratory and descriptive. The subtitle to Supplementary Table S3 further clarifies that balanced accuracy and the prevalence-weighted one-vs-rest AUC were not reported when one or more outcome classes were absent and that confidence intervals were not calculated because of sparse events within several subgroups.

The Discussion similarly treats these subgroup findings as contextual summaries of model behavior rather than as evidence that the model is more or less valid for specific disease categories.

**14. Results: binary sensitivity analysis**
**Point 23:** The binary sensitivity analysis is helpful, but the AUC confidence interval is wide, reflecting limited deceased events.
 The binary sensitivity analysis should be retained, but its interpretation should emphasize that discrimination was moderate and uncertain because of the small number of mortality events.
**Response 23:** We thank the reviewer for this important clarification. We agree that the binary deceased-versus-recovered sensitivity analysis should be retained but interpreted cautiously because only 17 deceased episodes were available and the resulting AUC confidence interval was wide.

We have therefore revised the Results to emphasize both the moderate discrimination and the associated uncertainty (Lines 361–364):

“In the binary deceased-versus-recovered sensitivity analysis, discrimination was moderate, with an AUC of 0.726 (95% CI, 0.585–0.839). The wide confidence interval indicated substantial uncertainty associated with the limited number of deceased episodes (Supplementary Table S5; Supplementary Fig S2).”

Supplementary Table S5 reports the corresponding binary analysis cohort and performance estimates, comprising 17 deceased and 184 recovered hospitalization episodes.

We have also revised the Discussion to avoid interpreting the binary analysis as evidence of stable mortality classification and instead emphasize the limited precision resulting from the small number of deceased events. It now reads (Lines 447–452):

“The binary deceased-versus-recovered sensitivity analysis provided a complementary assessment after ongoing episodes were excluded. Discrimination was moderate, but the wide confidence interval reflected substantial uncertainty associated with the limited number of deceased episodes. Calibration measures also indicated overestimation of deceased-outcome probabilities. Accordingly, this analysis should be interpreted cautiously as a sensitivity analysis rather than as evidence of stable mortality classification across alternative outcome formulations”

**15. Discussion: opening interpretation**
**Point 24:** The opening paragraph of the Discussion should be toned down. The current wording risks overstating the model’s clinical reliability. I recommend emphasizing that the model retained predictive signal overall but had variable performance across outcome classes, with weakest performance for deceased cases.
**Response 24:** We thank the reviewer for this important suggestion. We agree that the opening interpretation of the Discussion was too strong and could imply greater clinical reliability and transportability than are supported by the external validation results.

We have therefore revised the opening paragraph to emphasize that the model retained discriminatory information overall while also acknowledging its modest improvement over the majority-class benchmark, variation in performance across outcome classes and hospitals, and the weaker and less precise performance for deceased episodes. The revised Discussion now states (Lines 368–379):

“This study evaluated a previously developed random forest model for classifying hospitalization outcomes in Asian elephants using temporally distinct 2025 data from three elephant hospitals in Thailand. The frozen model was applied without refitting, recalibration, or updating, comprising temporal validation at the original development center and institutional validation at two additional hospitals. The model retained discriminatory information at the cohort level, but overall accuracy was only modestly higher than the recovered-only majority-class benchmark, and performance varied across outcome classes and hospitals. Classification performance was stronger for ongoing and recovered episodes than for deceased episodes, for which estimates were imprecise because only 17 deceased episodes were available. Calibration patterns also differed across outcome classes. These findings indicate that model performance was context dependent and should be interpreted cautiously, particularly for mortality-related classification.”

We also removed the previous broader statements that discrimination and calibration were “broadly acceptable” and that transportability was established. The revised wording is intended to distinguish evidence of retained classification signal from evidence of clinical reliability or readiness for implementation.

**16. Discussion: deceased outcome**
**Point 25:** The manuscript appropriately explains that lower deceased-class performance may reflect class imbalance and clinical heterogeneity.
 The authors should also acknowledge that the predictor set may be clinically insufficient for mortality classification. Age, sex, broad disease category and length of stay do not capture disease severity, physiological compromise, referral delay, treatment intensity or comorbidities.

The discussion of poorer deceased-class performance is appropriate but should more explicitly acknowledge that the predictor set may be clinically insufficient for mortality classification. Important mortality-related factors such as disease severity, physiological status, referral delay, treatment intensity, comorbidities and clinician-assessed prognosis were not included.
**Response 25:** We thank the reviewer for this important point. We agree that the poorer deceased-class performance should not be attributed only to class imbalance or heterogeneity in mortality pathways. The predictor set itself may also be clinically insufficient for distinguishing mortality outcomes because the frozen model included only age, sex, primary disease category, and length of stay.

We have therefore revised the Discussion to acknowledge explicitly the clinical information not represented in the model and the implications for mortality classification (Lines 390–402):

“Across the multiclass outcome framework, model performance differed by outcome category, with stronger classification performance for recovered and ongoing episodes than for deceased episodes. The deceased outcome was the least frequent class in the external validation cohort, which limited the precision of mortality-focused performance estimates. In addition to class imbalance, the predictor set may have been clinically insufficient for mortality classification. The model included only age, sex, broad primary disease category, and length of stay and therefore did not capture potentially important mortality-related information such as disease severity, physiological status, referral delay, treatment intensity, comorbidities, or clinician-assessed prognosis. These unmeasured dimensions may contribute to heterogeneity among deceased episodes and may partly explain the lower performance observed for this class. Mortality-related classification should therefore be interpreted cautiously, and future models may require richer clinical and longitudinal information to distinguish deceased outcomes more reliably.”

**17. Discussion: hospital heterogeneity**
**Point 26:** The discussion of hospital heterogeneity is thoughtful and important. However, key operational differences between hospitals, especially the meaning of ongoing care and the availability of inpatient capacity, should also be described earlier in the Methods or Results so readers can interpret the validation setting before reaching the Discussion.
**Response 26:** We thank the reviewer for this helpful suggestion. We agree that key operational differences between hospitals should be described before the Discussion so that readers can interpret the validation setting and hospital-specific performance in the appropriate clinical context.

We have therefore expanded the Study design and data sources section of the Methods to describe differences in referral structure, inpatient capacity, mobile care, and follow-up practices across the participating hospitals (Lines 137–144):

“Operational characteristics were described based on information provided by veterinarians at the participating hospitals and differed across hospitals in referral structure, inpatient capacity, and follow-up practices. Hospital A relied more heavily on mobile field-based care and had more limited inpatient capacity, whereas Hospitals B and C functioned as regional referral centers within the NEI network and had greater inpatient capacity. These differences were relevant to the interpretation of ongoing status because active veterinary management at the administrative cutoff did not necessarily imply continued physical hospitalization and could include home-based or mobile follow-up according to local hospital practice.”

The outcome-definition subsection (Lines 155–167) of the Methods also now defines ongoing status explicitly as active veterinary management at the administrative cutoff of 31 December 2025 and clarifies that this could include continued home-based or mobile follow-up rather than physical hospitalization alone.

**18. Discussion: calibration**
**Point 27:** The calibration discussion should be tempered because decile-based calibration may be unstable for rare classes. The authors should avoid strong claims about clinically interpretable probabilities unless calibration metrics support this.
**Response 27:** We thank the reviewer for this important caution. We agree that the previous Discussion overstated the calibration findings, particularly by suggesting that the model retained a “clinically interpretable probability structure” based primarily on graphical calibration. This interpretation is not sufficiently supported given the class-specific calibration deviations and the limited number of deceased episodes.

We have therefore removed the previous broad statements that predicted probabilities generally tracked observed event rates and that the model retained clinically interpretable probability structure. The revised Discussion now emphasizes the class-specific calibration patterns and associated uncertainty (Lines 423–435):

“Calibration analysis provided complementary information to discrimination, but the findings did not support a uniform conclusion of good calibration across outcome classes. Quantitative calibration measures indicated overall overestimation of deceased-outcome probabilities and underestimation of recovered-outcome probabilities, whereas calibration for the ongoing class was closer to the ideal values. The deceased class also showed poorer probabilistic performance than the prevalence-based reference model. Because only 17 deceased episodes were available, both the graphical calibration pattern and quantitative estimates for this class should be interpreted cautiously. This distinction is important because many machine learning studies in veterinary and biomedical medicine emphasize discrimination metrics while giving less attention to calibration (14,30,31). However, discrimination alone does not indicate whether predicted probabilities are numerically aligned with observed outcomes (16,22). Accordingly, the predicted probabilities in the present study should not be regarded as clinically calibrated estimates without further validation and, where appropriate, recalibration.”

The corresponding quantitative calibration estimates and 95% confidence intervals are reported in the Results and Supplementary Table S2.

We therefore no longer characterize calibration as broadly acceptable or the predicted probabilities as clinically interpretable without qualification.

**19. Limitations**
**Point 28:** The limitations section already includes several important caveats: small deceased-case count, dynamic ongoing category, partial institutional validation, use of length of stay, and no recalibration.
 Add limitation 1: Repeated admissions. The manuscript refers to admissions, not individual elephants. If some elephants had multiple admissions, observations may not be independent.
 Please report whether the 237 admissions represent 237 unique elephants or whether some animals contributed multiple admissions. If repeated admissions occurred, the authors should describe how this was handled analytically, particularly in bootstrap confidence intervals.
 Add limitation 2: Majority-class baseline. Overall accuracy is close to the recovered-only baseline and should be interpreted cautiously.
Add limitation 3: Administrative/workflow effects. Length of stay and ongoing status may reflect hospital workflow, not only biological or clinical prognosis.
**Response 28:** We thank the reviewer for these important suggestions. We have revised the Limitations to address repeated admissions, the majority-class benchmark, and the potential influence of administrative and workflow factors more explicitly.

The final validation cohort comprised 237 hospitalization episodes contributed by 215 elephants. Twenty-two elephants contributed more than one eligible hospitalization episode, accounting for 44 episodes. Because episode-level observations were therefore not completely independent, confidence intervals were estimated using bootstrap resampling clustered by elephant. We also performed a sensitivity analysis retaining only the first eligible hospitalization episode per elephant, which produced similar overall performance estimates.

We also explicitly contextualized overall accuracy against the recovered-only majority-class benchmark and clarified that the ongoing category and length of stay may reflect hospital workflow and administrative practices in addition to the patient’s clinical course.

The revised Limitations now state (Lines 453–483):

“Several limitations should be considered. First, the number of deceased cases in the external dataset was relatively small, which reduced statistical precision for mortality-focused analyses and widened uncertainty around class-specific performance estimates. Second, recovered episodes constituted the majority of the validation cohort, and overall accuracy was only modestly higher than that of the recovered-only majority-class benchmark. Overall accuracy should therefore be interpreted together with balanced accuracy, class-specific performance, probabilistic error, and calibration rather than as a standalone measure of model performance. Third, twenty-two elephants contributed more than one eligible hospitalization episode, accounting for 44 episodes, so episode-level observations were not completely independent. To account for this clustering, confidence intervals were estimated using bootstrap resampling clustered by elephant, and a sensitivity analysis retaining only the first eligible hospitalization episode per elephant produced similar overall performance estimates. Nevertheless, some within-elephant dependence may remain in the episode-level analysis. Fourth, the ongoing category reflects a dynamic clinical and administrative status rather than a final endpoint, and its interpretation may differ across hospitals depending on workflow, monitoring practices, follow-up arrangements, and discharge timing. Fifth, although the validation dataset incorporated new institutions, one participating hospital was also the original development center, albeit from a later time period. Accordingly, this study represents external validation under temporal shift and partial institutional variation rather than a fully independent multi-institutional validation in which all centers are entirely external to model development. Sixth, the frozen model included length of stay, which is generated during hospitalization and may reflect not only the patient’s clinical course but also discharge practices, inpatient capacity, follow-up arrangements, ongoing-care definitions, and the administrative timing of outcome ascertainment. Retaining this variable was necessary to preserve the original predictor set during external validation, but its inclusion limits interpretation of the model as an admission-time prognostic tool. In the exploratory analysis, the model excluding length of stay showed lower overall discrimination and balanced accuracy and did not classify any ongoing episodes. Although this separately fitted model was exploratory, these findings suggest that hospitalization-duration information contributed to classification performance. Finally, the model was applied without recalibration or updating. This preserves the methodological purpose of external validation, but the potential effects of local recalibration or model updating were not evaluated in the present study.”

**20. Conclusion**
**Point 29:** The conclusion is clear but slightly too confident. It should be more specific about what was validated and more cautious about clinical implementation. The conclusion should avoid implying readiness for clinical use. It should emphasize that performance was strongest for recovered and ongoing cases, weaker for deceased cases, and limited by the use of length of stay.
**Response 29:** We thank the reviewer for this important suggestion. We agree that the previous Conclusion was too confident and could be interpreted as implying greater clinical readiness than is supported by the external validation results.

We have therefore revised the Conclusion to specify the validation design, emphasize the stronger performance for ongoing and recovered episodes relative to deceased episodes, acknowledge the limitation imposed by the inclusion of length of stay, and avoid implying readiness for routine clinical implementation. The revised Conclusion now states (Lines 494–506):

“This study evaluated a previously developed frozen random forest model for classifying hospitalization outcomes in Asian elephants through temporal validation at the original development center and institutional validation at two additional elephant hospitals in Thailand. The model retained discriminatory information in the 2025 validation cohort, but overall accuracy was only modestly higher than the recovered-only majority-class benchmark, and classification performance was stronger for ongoing and recovered episodes than for deceased episodes. Because length of stay was included as a predictor, the model should be interpreted as a hospitalization-course classification model using information accumulated during care rather than as an admission-time prognostic tool. The limited number of deceased episodes and the observed class-specific calibration differences further warrant cautious interpretation. These findings do not establish readiness for routine clinical use and support further prospective evaluation, with local recalibration or model updating considered where appropriate before implementation.”

We also removed the previous broader statements that the model showed “useful overall performance,” that predicted probabilities remained broadly aligned with observed outcomes, and that the binary sensitivity analysis provided additional support for the stability of the findings. The revised Conclusion is intended to reflect the scope and limitations of the present validation more precisely.

**Point 30:** Minor wording and consistency edits
Mixed spelling: center/centre; harmonised/harmonized: Choose either US or UK spelling and use consistently.
“Macro-weighted” AUC: Define the exact averaging method.
“Acknowledgement”: Use “Acknowledgments” if following PLOS style.
Reference typo: “Veterinary Internal Medicne”: Correct to “Veterinary Internal Medicine”.
Figure 2 caption lacks final punctuation: Add a full stop.
Repeated phrase “useful overall performance”: Replace some instances with more specific metric-based wording.
Hospital anonymity unclear: Clarify whether Hospital A/B/C are intended to be anonymous, given locations and institutional affiliations are identifiable.
“Prediction model” language: Use “classification model” where discussing the current model, especially because length of stay is included.

**Response 30:** We thank the reviewer for these careful wording and consistency suggestions. We conducted a manuscript-wide review and addressed each of these points.

First, we standardized the manuscript to US English, including consistent use of “center” and “harmonized.” We also removed the ambiguous term “macro-weighted” AUC and replaced it throughout with “prevalence-weighted one-vs-rest AUC.” As clarified in the Methods, this measure was calculated as the weighted mean of the three class-specific one-vs-rest AUCs, using the observed prevalence of each outcome class as the weight.

We changed the section heading from “Acknowledgement” to “Acknowledgments” (Lines 508–511), corrected “Veterinary Internal Medicne” to “Veterinary Internal Medicine” in the reference list (Lines 587–590), and added the missing final punctuation to the Figure 2 caption (Lines 318–322).

We also removed or replaced repeated qualitative phrases such as “useful overall performance” with more specific descriptions of the observed performance and its limitations. In particular, the revised Abstract, Discussion, and Conclusion now distinguish retained discriminatory information from evidence of clinical reliability or readiness for routine implementation.

Regarding the hospital labels, the participating institutions are not intended to be anonymous because their locations and operating institutions are described in the manuscript. Hospitals A, B, and C are used solely for consistency and readability throughout the manuscript. We clarified this in the Methods by revising the description to state (Lines 119–122):

“The external validation dataset consisted of clinical records from hospitalization episodes active during the 2025 calendar year (1 January to 31 December 2025) across three elephant hospitals in Thailand, referred to throughout the manuscript as Hospitals A, B, and C for consistency of presentation.”

Finally, we reviewed terminology throughout the manuscript and use “classification model” when referring to the model evaluated in the present study. Broader terms such as “prediction” and “prognostic” are retained only where they refer to the wider literature or general methodological context rather than to the interpretation of the current hospitalization-course classification model.
