# Supplementary material for: External validation of a machine learning model for classifying hospitalization outcomes in Asian elephants across three elephant hospitals in Thailand
Source: PLoS One. 2026 Nov 11;21(11):e0362469. doi: 10.1371/journal.pone.0362469 (PMC13754164; doi:10.1371/journal.pone.0362469)
Supplement: Peer review history

# Peer review: Decision Letter 0

**DOI:** 10.1371/journal.pone.0362469.r001

**Authors:** Julio Souza

27 Jul 2026

Dear Dr. Kosaruk,

Thank you for submitting your manuscript to PLOS ONE. After careful consideration, we feel that it has merit but does not fully meet PLOS ONE’s publication criteria as it currently stands. Therefore, we invite you to submit a revised version of the manuscript that addresses the points raised during the review process.

==============================

Dear Sir, in light of the reviewers' comments,

I request that you address them so that we may proceed with the publication.

Best regards,

JCS

==============================

Please submit your revised manuscript by Sep 25 2026 11:59PM. If you will need more time than this to complete your revisions, please reply to this message or contact the journal office at plosone@plos.org. When you're ready to submit your revision, log on to https://www.editorialmanager.com/pone/ and select the 'Submissions Needing Revision' folder to locate your manuscript file.

- A letter that responds to each point raised by the academic editor and reviewer(s). You should upload this letter as a separate file labeled 'Response to Reviewers'.
- A marked-up copy of your manuscript that highlights changes made to the original version. You should upload this as a separate file labeled 'Revised Manuscript with Track Changes'.
- An unmarked version of your revised paper without tracked changes. You should upload this as a separate file labeled 'Manuscript'.

If you would like to make changes to your financial disclosure, please include your updated statement in your cover letter. Guidelines for resubmitting your figure files are available below the reviewer comments at the end of this letter.

If applicable, we recommend that you deposit your laboratory protocols in protocols.io to enhance the reproducibility of your results. Protocols.io assigns your protocol its own identifier (DOI) so that it can be cited independently in the future. For instructions see: https://journals.plos.org/plosone/s/submission-guidelines#loc-laboratory-protocols. Additionally, PLOS ONE offers an option for publishing peer-reviewed Lab Protocol articles, which describe protocols hosted on protocols.io. Read more information on sharing protocols at https://plos.org/protocols?utm_medium=editorial-email&utm_source=authorletters&utm_campaign=protocols.

As the corresponding author, your ORCID iD is verified in the submission system and will appear in the published article. PLOS supports the use of ORCID, and we encourage all coauthors to register for an ORCID iD and use it as well. Please encourage your coauthors to verify their ORCID iD within the submission system before final acceptance, as unverified ORCID iDs will not appear in the published article. *Only*  the individual author can complete the verification step; PLOS staff *cannot*  verify ORCID iDs on behalf of authors.

We look forward to receiving your revised manuscript.

Kind regards,

Julio Cesar de Souza, Ph.D.

Academic Editor

PLOS One

Journal Requirements:

When submitting your revision, we need you to address these additional requirements.

1. Please ensure that your manuscript meets PLOS ONE's style requirements, including those for file naming. The PLOS ONE style templates can be found at

https://journals.plos.org/plosone/s/file?id=wjVg/PLOSOne_formatting_sample_main_body.pdf and

https://journals.plos.org/plosone/s/file?id=ba62/PLOSOne_formatting_sample_title_authors_affiliations.pdf

2. Please note that PLOS One has specific guidelines on code sharing for submissions in which author-generated code underpins the findings in the manuscript. In these cases, we expect all author-generated code to be made available without restrictions upon publication of the work. Please review our guidelines at https://journals.plos.org/plosone/s/materials-and-software-sharing#loc-sharing-code and ensure that your code is shared in a way that follows best practice and facilitates reproducibility and reuse.

3. Please include captions for your Supporting Information files at the end of your manuscript, and update any in-text citations to match accordingly. Please see our Supporting Information guidelines for more information: http://journals.plos.org/plosone/s/supporting-information.

4. Thank you for stating the following financial disclosure:

"This study was partially supported by Chiang Mai University."

Please state what role the funders took in the study.  If the funders had no role, please state: "The funders had no role in study design, data collection and analysis, decision to publish, or preparation of the manuscript."

If this statement is not correct you must amend it as needed.

Please include this amended Role of Funder statement in your cover letter; we will change the online submission form on your behalf.

5. We note that there is identifying data in the Supporting Information file Supporting Information - Raw data and R codes. Due to the inclusion of these potentially identifying data, we have removed this file from your file inventory. Prior to sharing human research participant data, authors should consult with an ethics committee to ensure data are shared in accordance with participant consent and all applicable local laws.

Data sharing should never compromise participant privacy. It is therefore not appropriate to publicly share personally identifiable data on human research participants. The following are examples of data that should not be shared:

-Name, initials, physical address

-Ages more specific than whole numbers

-Internet protocol (IP) address

-Specific dates (birth dates, death dates, examination dates, etc.)

-Contact information such as phone number or email address

-Location data

-ID numbers that seem specific (long numbers, include initials, titled “Hospital ID”) rather than random (small numbers in numerical order)

Data that are not directly identifying may also be inappropriate to share, as in combination they can become identifying. For example, data collected from a small group of participants, vulnerable populations, or private groups should not be shared if they involve indirect identifiers (such as sex, ethnicity, location, etc.) that may risk the identification of study participants.

Additional guidance on preparing raw data for publication can be found in our Data Policy (https://journals.plos.org/plosone/s/data-availability#loc-human-research-participant-data-and-other-sensitive-data) and in the following article: http://www.bmj.com/content/340/bmj.c181.long.

Please remove or anonymize all personal information age, ensure that the data shared are in accordance with participant consent, and re-upload a fully anonymized data set. Please note that spreadsheet columns with personal information must be removed and not hidden as all hidden columns will appear in the published file.

6. If the reviewer comments include a recommendation to cite specific previously published works, please review and evaluate these publications to determine whether they are relevant and should be cited. There is no requirement to cite these works unless the editor has indicated otherwise.

Additional Editor Comments:

Dear Sir, in light of the reviewers' comments,

I request that you address them so that we may proceed with the publication.

Best regards,

JCS

[Note: HTML markup is below. Please do not edit.]

Reviewers' comments:

Reviewer's Responses to Questions

**Comments to the Author**

1. Is the manuscript technically sound, and do the data support the conclusions?

Reviewer #1: Partly

Reviewer #2: Yes

**********

2. Has the statistical analysis been performed appropriately and rigorously?

Reviewer #1: No

Reviewer #2: Yes

**********

3. Have the authors made all data underlying the findings in their manuscript fully available?

The PLOS Data policy

Reviewer #1: Yes

Reviewer #2: Yes

**********

4. Is the manuscript presented in an intelligible fashion and written in standard English?

Reviewer #1: Yes

Reviewer #2: Yes

**********

Reviewer #1: Manuscript: External validation of a machine learning model for clinical outcome classification in hospitalized Asian elephants across three elephant hospitals in Thailand

Review recommendation

Recommended decision: Major revision

Overall assessment: Potentially publishable and valuable, but the claims need to be narrowed and the reporting made more transparent.

Main interpretive concern: Length of stay is included as a predictor, so the model should not be framed as an admission-time prognostic tool.

Main reporting concern: Recovered cases dominate the cohort, deceased events are sparse, and the ongoing outcome is not a stable final endpoint.

Overall comment: This is a worthwhile, clearly written study that addresses an important gap: external validation of a clinical prediction/classification model in a wildlife hospital context. The validation design is appropriate in principle because the authors apply a frozen previously developed random forest model to temporally distinct 2025 records from three elephant hospitals without refitting or recalibration. However, substantial revision is needed to ensure the language, claims and performance interpretation match what the model can actually support.

Highest-priority issues

Priority 1. Reframe the model: Because length of stay is included as a predictor, avoid presenting the model as an admission-time prognostic tool.

Priority 2. Benchmark against the majority class: Recovered cases are 78.9% of the cohort and overall accuracy is 0.797, so accuracy alone is not impressive without comparison to a recovered-only classifier.

Priority 3. Be cautious about mortality classification: There are only 17 deceased cases; deceased-class performance and calibration will be uncertain.

Priority 4. Define ongoing clearly: Ongoing is a dynamic state and may reflect hospital workflow, mobile follow-up or administrative status rather than a final clinical outcome.

Priority 5. Clarify external validation type: One hospital is the original development centre, so this is temporal validation plus partial institutional validation, not a fully independent multi-centre validation.

Priority 6. Report exclusions and repeated admissions: The authors should provide a flow summary and clarify whether the 237 admissions represent unique elephants.

Priority 7. Strengthen calibration reporting: Visual decile plots are useful but insufficient, especially with sparse death events.

Section comments and suggested edits

1. Title and short title

The current title is broadly accurate, but it may imply a general clinical prediction or admission-time prognosis model. Because length of stay is included, the title should make clear that this is hospitalization outcome classification.

The title should clarify that the model classifies hospitalization outcomes rather than predicting prognosis at admission. This distinction matters because one of the predictors is length of stay, which is not known at admission and may be affected by the clinical course and hospital workflow.

2. Abstract

The abstract should provide more quantitative information. Phrases such as “retained useful overall performance” and “acceptable agreement” are difficult to interpret without the sample size, outcome distribution and performance metrics. Please report N = 237 admissions, the class distribution, the main multiclass metrics, and the binary sensitivity analysis AUC. The abstract should also state that performance was weaker for deceased cases and that mortality-related conclusions are limited by sparse death events.

Add to abstract: N = 237 admissions; deceased = 17, ongoing = 33, recovered = 187; accuracy = 0.797; macro/balanced accuracy = 0.741; multiclass AUC = 0.862; log loss = 0.564; binary deceased-versus-recovered AUC = 0.722, 95% CI 0.594–0.834.

3. Introduction

The introduction provides a strong rationale for external validation and explains why elephant hospital data may differ by institution, time period, case mix and clinical workflow.

The introduction uses prognostic language quite broadly. That is understandable, but this particular model includes length of stay, so the manuscript needs to distinguish admission-time prognosis from outcome classification using information accumulated during hospitalization.

Please distinguish between admission-time prognosis and outcome classification using hospital-course information. This is important because the original model includes length of stay, which is not available at admission and may be influenced by the outcome and hospital workflow.

Suggested wording- Because the original model included length of stay, the present validation should be interpreted as an evaluation of hospitalization outcome classification using routinely recorded demographic, diagnostic and hospitalization-duration variables, rather than as validation of an admission-time prognostic tool.

The objective should also acknowledge that one validation hospital was the original development centre.

4. Methods: study design and data sources

The authors appropriately state that the model was applied without refitting, recalibration or updating. This is important and should remain prominent.

Because one hospital was the original development centre, this is not a fully independent multi-institutional validation. It is a temporal validation at the original centre plus institutional validation at two additional hospitals.

Please clarify throughout the manuscript that this is temporal validation with partial institutional external validation, rather than a fully independent validation in which all hospitals were external to the original model-development setting.

5. Methods: inclusion criteria and exclusions

The manuscript states which records were eligible but does not report how many 2025 records were screened or excluded. This is a key transparency issue because exclusions could bias validation performance.

Please provide a record flow summary showing the number of admissions screened at each hospital, the number excluded, and reasons for exclusion. This could be included in the Results or as a supplementary flow diagram.

Information needed: Total 2025 admissions available by hospital; number excluded for missing age, sex, disease category, outcome, admission date or discharge date; and the final analytic dataset by hospital.

Suggested wording - A total of X admissions were screened across the three hospitals. Of these, X were excluded because of missing or unclear data, including X with missing age, X with missing disease category, X with missing outcome status, and X with unclear admission or discharge dates. The final analytic dataset included 237 admissions.

6. Methods: outcome definitions

The “ongoing” outcome is central to the model but is not a stable final endpoint. It needs clearer operational definition. The Discussion notes that ongoing status may include home-based or mobile follow-up, but this belongs in the Methods as well.

Please define the ongoing category more clearly. It is currently unclear whether ongoing consistently means physically hospitalized, still under active veterinary management, receiving mobile follow-up, or not yet administratively discharged. Because this category may reflect hospital workflow as much as clinical status, this definition is essential for interpreting the model.

Also needed - The exact data extraction date, whether ongoing status was determined consistently across hospitals, and whether ongoing cases were later resolved after the extraction date.

7. Methods: predictor variables

Length of stay is included as a predictor. This is the most important methodological and interpretive issue in the manuscript.

Length of stay is not available at admission and may be directly affected by outcome, discharge policy, case severity, hospital capacity, follow-up practices and data extraction timing. For ongoing cases, length of stay is censored at 31 December 2025, making it particularly tied to outcome definition.

The inclusion of length of stay should be more explicitly discussed as a potential source of outcome-related information. Because length of stay is determined during hospitalization and may be affected by discharge practices, clinical deterioration, ongoing care definitions and data extraction timing, the model should not be described as an admission-time prognostic tool. Please revise the manuscript accordingly and consider an additional sensitivity analysis using only predictors available at admission.

If possible, evaluate or develop a model using only age, sex and primary disease category. Even if performance decreases, it would help distinguish true admission-time prognostic performance from hospital-course classification.

Suggested wording - Because length of stay was included as a predictor, the model incorporates information generated during hospitalization and should not be interpreted as an admission-time prognostic tool. Length of stay may also reflect hospital workflow, discharge policies and follow-up practices, in addition to patient condition.

8. Methods: preprocessing and model application

The manuscript says categorical predictors were one-hot encoded and zero-variance predictors were removed. It should be explicit whether this was done using the frozen preprocessing recipe from the development dataset or recalculated in the validation data.

Please clarify whether zero-variance removal was part of the frozen preprocessing recipe derived from the development dataset, or whether it was recalculated in the external dataset. For strict external validation, factor levels, dummy-variable structure and preprocessing steps should be defined from the development data and applied unchanged to the validation data.

9. Methods: performance evaluation

Overall accuracy may be misleading because recovered cases dominate the cohort. A model that predicted all cases as recovered would already achieve approximately 78.9% accuracy, while the reported accuracy is 79.7%.

Because recovered cases comprised 78.9% of the validation cohort, overall accuracy should be interpreted against the no-information rate or majority-class classifier. Please report the majority-class baseline accuracy and emphasize balanced accuracy, class-specific recall/precision, log loss and calibration rather than accuracy alone.

The phrase “macro-weighted multiclass AUC” is ambiguous. Macro-averaged and weighted-averaged metrics are different.

Please clarify whether the reported multiclass AUC was macro-averaged, weighted macro-averaged, or calculated using another multiclass method. The current phrase “macro-weighted” is ambiguous.

Calibration is currently assessed visually using deciles. This is useful, but quantitative calibration metrics would strengthen the paper.

Calibration assessment would be strengthened by reporting quantitative calibration metrics, such as Brier score, calibration-in-the-large, calibration slope or expected calibration error, with confidence intervals where feasible. If these cannot be estimated reliably because of sparse events, this should be stated.

10. Results: external validation cohort

Table 1 is useful and clearly shows important differences in age, length of stay, disease distribution and outcomes across hospitals.

The Results should make the implications of the outcome distribution more explicit. Hospital A had no ongoing cases, Hospital C contributed most ongoing cases, and deceased events were sparse overall.

Please more explicitly describe how outcome distribution differed across hospitals. The absence of ongoing cases in Hospital A and the concentration of ongoing cases in Hospital C are important because they affect hospital-specific metrics and interpretation of transportability.

11. Results: multiclass performance

The main text gives overall metrics but leaves important class-specific metrics in the supplementary material. Deceased-class recall, precision and F1 score should be in the main Results because mortality classification is clinically important.

Please include key class-specific metrics in the main Results text, particularly recall, precision and F1 score for the deceased class. Readers should not need to consult supplementary material to evaluate whether the model identifies deceased cases adequately.

Suggested wording. - Although overall discrimination was acceptable, deceased-class performance was lower than recovered and ongoing performance, with reduced sensitivity and precision. This indicates that the model was less reliable for identifying mortality outcomes than for classifying recovered or ongoing cases.

When reporting accuracy, explicitly compare it with the recovered-only baseline. This is one of the most important interpretive points in the review.

12. Results: calibration

The manuscript says predicted probabilities generally corresponded with observed event rates. This should be more cautious, especially for deceased cases, where there are only 17 events.

The calibration results should be interpreted more cautiously, especially for deceased cases. With only 17 deaths, decile-based observed mortality rates may be unstable. Please avoid broad statements that calibration was acceptable unless supported by quantitative calibration statistics or confidence intervals.

13. Results: disease-stratified performance

The disease-stratified analyses are useful descriptively, but some disease categories have small numbers or lack one or more outcome classes.

The disease-stratified analyses should remain clearly labelled as descriptive and exploratory. The authors should avoid implying that the model is more or less valid for particular disease categories unless there are sufficient numbers of all outcome classes.

14. Results: binary sensitivity analysis

The binary sensitivity analysis is helpful, but the AUC confidence interval is wide, reflecting limited deceased events.

The binary sensitivity analysis should be retained, but its interpretation should emphasize that discrimination was moderate and uncertain because of the small number of mortality events.

15. Discussion: opening interpretation

The opening paragraph of the Discussion should be toned down. The current wording risks overstating the model’s clinical reliability. I recommend emphasizing that the model retained predictive signal overall but had variable performance across outcome classes, with weakest performance for deceased cases.

16. Discussion: deceased outcome

The manuscript appropriately explains that lower deceased-class performance may reflect class imbalance and clinical heterogeneity.

The authors should also acknowledge that the predictor set may be clinically insufficient for mortality classification. Age, sex, broad disease category and length of stay do not capture disease severity, physiological compromise, referral delay, treatment intensity or comorbidities.

The discussion of poorer deceased-class performance is appropriate but should more explicitly acknowledge that the predictor set may be clinically insufficient for mortality classification. Important mortality-related factors such as disease severity, physiological status, referral delay, treatment intensity, comorbidities and clinician-assessed prognosis were not included.

17. Discussion: hospital heterogeneity

The discussion of hospital heterogeneity is thoughtful and important. However, key operational differences between hospitals, especially the meaning of ongoing care and the availability of inpatient capacity, should also be described earlier in the Methods or Results so readers can interpret the validation setting before reaching the Discussion.

18. Discussion: calibration

The calibration discussion should be tempered because decile-based calibration may be unstable for rare classes. The authors should avoid strong claims about clinically interpretable probabilities unless calibration metrics support this.

19. Limitations

The limitations section already includes several important caveats: small deceased-case count, dynamic ongoing category, partial institutional validation, use of length of stay, and no recalibration.

Add limitation 1: Repeated admissions. The manuscript refers to admissions, not individual elephants. If some elephants had multiple admissions, observations may not be independent.

Please report whether the 237 admissions represent 237 unique elephants or whether some animals contributed multiple admissions. If repeated admissions occurred, the authors should describe how this was handled analytically, particularly in bootstrap confidence intervals.

Add limitation 2: Majority-class baseline. Overall accuracy is close to the recovered-only baseline and should be interpreted cautiously.

Add limitation 3: Administrative/workflow effects. Length of stay and ongoing status may reflect hospital workflow, not only biological or clinical prognosis.

20. Conclusion

The conclusion is clear but slightly too confident. It should be more specific about what was validated and more cautious about clinical implementation. The conclusion should avoid implying readiness for clinical use. It should emphasize that performance was strongest for recovered and ongoing cases, weaker for deceased cases, and limited by the use of length of stay.

Minor wording and consistency edits

Mixed spelling: center/centre; harmonised/harmonized: Choose either US or UK spelling and use consistently.

“Macro-weighted” AUC: Define the exact averaging method.

“Acknowledgement”: Use “Acknowledgments” if following PLOS style.

Reference typo: “Veterinary Internal Medicne”: Correct to “Veterinary Internal Medicine”.

Figure 2 caption lacks final punctuation: Add a full stop.

Repeated phrase “useful overall performance”: Replace some instances with more specific metric-based wording.

Hospital anonymity unclear: Clarify whether Hospital A/B/C are intended to be anonymous, given locations and institutional affiliations are identifiable.

“Prediction model” language: Use “classification model” where discussing the current model, especially because length of stay is included.

Reviewer #2: I would like to congratulate the authors on this important, well-conducted, and well-written study. The data presented are remarkable and will be highly valuable to other institutions, including those beyond elephant-focused facilities. I have only two minor suggestions: please include the version of the R software used in the Methods section and clarify that ethical review was waived for this study.

**********

what does this mean?). If published, this will include your full peer review and any attached files.

If you choose “no”, your identity will remain anonymous but your review may still be made public.

**Do you want your identity to be public for this peer review?**  For information about this choice, including consent withdrawal, please see our Privacy Policy

Reviewer #1: No

Reviewer #2: **Yes:**  Sofia Silva La Rocca de Freitas

**********

[NOTE: If reviewer comments were submitted as an attachment file, they will be attached to this email and accessible via the submission site. Please log into your account, locate the manuscript record, and check for the action link "View Attachments". If this link does not appear, there are no attachment files.]

To ensure your figures meet our technical requirements, please review our figure guidelines: https://journals.plos.org/plosone/s/figures

You may also use PLOS’s free figure tool, NAAS, to help you prepare publication quality figures: https://journals.plos.org/plosone/s/figures#loc-tools-for-figure-preparation.

NAAS will assess whether your figures meet our technical requirements by comparing each figure against our figure specifications.

# Author comment: Author response to Decision Letter 1

**DOI:** 10.1371/journal.pone.0362469.r002

27 Sep 2026

We sincerely thank the Editor and Reviewers for their careful and constructive comments. We have revised the manuscript extensively in response to all points raised. A detailed point-by-point response to Reviewer #1 and Reviewer #2, including the corresponding revisions and manuscript line numbers, has been provided in the uploaded Response to Reviewers files. All reviewer comments have been addressed in the revised manuscript.

Attachment Submitted filename: Response to Reviewer 1.docx

# Peer review: Decision Letter 1

**DOI:** 10.1371/journal.pone.0362469.r003

**Authors:** Julio Souza

27 Oct 2026

External validation of a machine learning model for classifying hospitalization outcomes in Asian elephants across three elephant hospitals in Thailand

PONE-D-26-20496R1

Dear Dr. Kosaruk,

We’re pleased to inform you that your manuscript has been judged scientifically suitable for publication and will be formally accepted for publication once it meets all outstanding technical requirements.

Within one week, you’ll receive an e-mail detailing the required amendments. When these have been addressed, you’ll receive a formal acceptance letter and your manuscript will be scheduled for publication.

An invoice will be generated when your article is formally accepted. Please note, if your institution has a publishing partnership with PLOS and your article meets the relevant criteria, all or part of your publication costs will be covered. Please make sure your user information is up-to-date by logging into Editorial Manager at Editorial Manager® and clicking the ‘Update My Information' link at the top of the page. For questions related to billing, please contact billing support.

If your institution or institutions have a press office, please notify them about your upcoming paper to help maximize its impact. If they’ll be preparing press materials, please inform our press team as soon as possible -- no later than 48 hours after receiving the formal acceptance. Your manuscript will remain under strict press embargo until 2 pm Eastern Time on the date of publication. For more information, please contact onepress@plos.org.

Kind regards,

Julio Cesar de Souza, Ph.D.

Academic Editor

PLOS One

Additional Editor Comments (optional):

Considering the feedback received, I am in favor of publishing the paper.

Best Regards

Reviewers' comments:

Reviewer's Responses to Questions

**Comments to the Author**

Reviewer #1: (No Response)

Reviewer #2: All comments have been addressed

**********

2. Is the manuscript technically sound, and do the data support the conclusions?

Reviewer #1: Yes

Reviewer #2: Yes

**********

3. Has the statistical analysis been performed appropriately and rigorously?

Reviewer #1: Yes

Reviewer #2: Yes

**********

4. Have the authors made all data underlying the findings in their manuscript fully available?

The PLOS Data policy

Reviewer #1: Yes

Reviewer #2: Yes

**********

5. Is the manuscript presented in an intelligible fashion and written in standard English?

Reviewer #1: Yes

Reviewer #2: Yes

**********

Reviewer #1: Minor revision.

The authors have responded thoroughly and constructively to the review. The additional analyses are appropriate, and the revised manuscript presents a substantially more cautious and scientifically defensible interpretation.

I would not request further major statistical analyses. The limitations of the model are now adequately demonstrated and discussed.

I would ask only for clarification of the intended timing and potential practical use of the model, whether individual elephants occurred in both development and validation datasets.

Reviewer #2: (No Response)

**********

what does this mean?). If published, this will include your full peer review and any attached files.

If you choose “no”, your identity will remain anonymous but your review may still be made public.

**Do you want your identity to be public for this peer review?**  For information about this choice, including consent withdrawal, please see our Privacy Policy

Reviewer #1: No

Reviewer #2: **Yes:**  Sofia Silva La Rocca de Freitas

**********

# Editor report: Acceptance letter

**DOI:** 10.1371/journal.pone.0362469.r004

**Authors:** Julio Souza

PONE-D-26-20496R1

PLOS One

Dear Dr. Kosaruk,

I'm pleased to inform you that your manuscript has been deemed suitable for publication in PLOS One. Congratulations! Your manuscript is now being handed over to our production team.

At this stage, our production department will prepare your paper for publication. This includes ensuring the following:

* All references, tables, and figures are properly cited

* All relevant supporting information is included in the manuscript submission,

* There are no issues that prevent the paper from being properly typeset

You will receive further instructions from the production team, including instructions on how to review your proof when it is ready. Please keep in mind that we are working through a large volume of accepted articles, so please give us a few days to review your paper and let you know the next and final steps.

Lastly, if your institution or institutions have a press office, please let them know about your upcoming paper now to help maximize its impact. If they'll be preparing press materials, please inform our press team within the next 48 hours. Your manuscript will remain under strict press embargo until 2 pm Eastern Time on the date of publication. For more information, please contact onepress@plos.org.

You will receive an invoice from PLOS for your publication fee after your manuscript has reached the completed accept phase. If you receive an email requesting payment before acceptance or for any other service, this may be a phishing scheme. Learn how to identify phishing emails and protect your accounts at https://explore.plos.org/phishing.

If we can help with anything else, please email us at customercare@plos.org.

Thank you for submitting your work to PLOS One and supporting open access.

Kind regards,

PLOS One Editorial Office Staff

on behalf of

Dr. Julio Cesar de Souza

Academic Editor

PLOS One
